# An engineered high affinity Fbs1 carbohydrate binding protein for selective capture of N-glycans and N-glycopeptides

**DOI:** 10.1038/ncomms15487

**Published:** 2017-05-23

**Authors:** Minyong Chen, Xiaofeng Shi, Rebecca M. Duke, Cristian I. Ruse, Nan Dai, Christopher H. Taron, James C. Samuelson

**Affiliations:** 1 New England Biolabs Inc., 240 County Road, Ipswich, Massachusetts 01938, USA

## Abstract

A method for selective and comprehensive enrichment of N-linked glycopeptides was developed to facilitate detection of micro-heterogeneity of N-glycosylation. The method takes advantage of the inherent properties of Fbs1, which functions within the ubiquitin-mediated degradation system to recognize the common core pentasaccharide motif (Man3GlcNAc2) of N-linked glycoproteins. We show that Fbs1 is able to bind diverse types of N-linked glycomolecules; however, wild-type Fbs1 preferentially binds high-mannose-containing glycans. We identified Fbs1 variants through mutagenesis and plasmid display selection, which possess higher affinity and improved recovery of complex N-glycomolecules. In particular, we demonstrate that the Fbs1 GYR variant may be employed for substantially unbiased enrichment of N-linked glycopeptides from human serum. Most importantly, this highly efficient N-glycopeptide enrichment method enables the simultaneous determination of N-glycan composition and N-glycosites with a deeper coverage (compared to lectin enrichment) and improves large-scale N-glycoproteomics studies due to greatly reduced sample complexity.

## Abstract (web summary)

Protein glycosylation is an essential post-translational modification which analysis is complicated by the diversity of glycan composition and heterogeneity at individual attachment sites. Here the authors describe a method to selectively enrich N-linked glycopeptides to facilitate the detection of micro-heterogeneity in N-glycosylation.

Glycosylation is one of the most common and structurally diverse forms of post-translational modification of eukaryotic proteins. It is estimated that >50% mammalian proteins possess some form of glycosylation and each mammalian cell may contain >10 million N- or O-linked glycans attached to proteins on its surface^1^. Glycans have been implicated in numerous important cellular functions, such as protein folding and trafficking, cell adhesion and signaling^2^. Additionally, numerous studies have reported correlations between changes in glycan structure and changes in human physiology (for example, aging, adolescence, menopause)^3^ or the onset of disease (for example, many cancers, rheumatoid arthritis, schizophrenia)^4^,^5^,^6^,^7^,^8^,^9^.

The glycan component of glycoproteins is inherently complex. This reflects the manner in which glycans are assembled and transferred to proteins. Glycan synthesis does not occur through precise interpretation of a genetic template, but instead, proceeds through the coordinated action of many biosynthetic enzymes^2^. Individual glycoproteins often exhibit heterogeneity both in the structural variety of the glycans they possess and the presence or absence of glycans at any potential glycan attachment site (glycosite). This glycan structural heterogeneity complicates both the analysis and physiological interpretation of complex glycoproteomes. Glycoproteomes are often analysed at the peptide level using proteomics methods. Typical bottom–up approaches for glycopeptide analysis require removal of glycans before analysis of the peptides by mass spectrometry (MS), which can be termed as deglycoproteomics^10^. Thus useful information about appended glycans is lost. Analysis of intact glycopeptides has become an increasingly important method in glycoproteomics^11^,^12^. In most intact glycopeptide analytical workflows, a glycoproteome is first digested with a protease (for example, trypsin) to generate a peptide pool. Because glycopeptides represent only a small fraction of this pool, enrichment is a necessary step to reduce the complexity of biological samples in methods designed for large-scale glycoproteomic studies^11^,^13^.

Three common approaches are applied to enrich glycopeptides: (1) Hydrazide-mediated methods involve covalent linkage to the glycan moiety. This enrichment method is primarily used for glycosite determination (deglycoproteomics)^14^ or more recently for analysis of intact sialylated glycopeptides^15^. (2) Hydrophilic interaction (HILIC) enrichment is a common approach for enriching glycans^16^. HILIC materials show broad specificity for glycans but do not discriminate between O- and N-linked glycomolecules and it may also enrich other hydrophilic biomolecules^17^. For effective glycopeptide isolation, HILIC enrichment requires a complementary chromatography step^18^,^19^. (3) The carbohydrate-binding properties of many lectins have been characterized and several have been employed for glycopeptide/glycoprotein enrichment or detection^20^. The specificity of a lectin may be quite narrow^21^ or relatively broad in the case of Concanavalin A (ConA), which recognizes a high-mannose structure. Furthermore, broad specificity lectins such as wheat germ agglutinin (WGA) bind to both N- and/or O-linked glycans^22^,^23^. A diverse set of lectins with selective affinities for specific carbohydrate epitopes has been used to investigate the human deglycoproteome^24^. To date, no single lectin has been shown to possess appropriate selectivity to analyse the N-linked glycoproteome^25^,^26^,^27^,^28^.

Ideally, unbiased enrichment of N-linked glycopeptides would be based on using a binding protein to specifically recognize the common core motif of N-glycans. An advantage of such an approach is that it would be possible to identify glycan composition/structure and glycosylation sites simultaneously after the N-glycopeptides are enriched. Robert Woods and co-workers have made progress towards this goal by engineering a catalytically inactive variant of PNGase F^29^,^30^. However, complete characterization of the R911 PNGase F variant has not been reported in the literature. When searching for a carbohydrate-binding protein that recognizes the common core of N-glycans, we looked to the mammalian cell for a protein with the desired inherent binding properties. Fbs1 (aka Fbx2, FBXO2, NFB42) is a component of the E3 ubiquitin ligase complex and is involved in the endoplasmic reticulum-associated degradation system for clearing of misfolded glycoproteins^31^,^32^. When N-linked glycoproteins misfold, Fbs1 protein specifically binds to the common N-glycan pentasaccharide core motif, Man3GlcNAc2 or M3N2 (see Fig. 1c for the structure), and brings ubiquitin ligase to the misfolded protein^33^. Although Fbs1 recognizes the M3N2 core of N-linked glycoproteins, it has been shown to preferentially bind to high-mannose-type N-glycans^34^.

Our objective to develop a method for N-glycopeptide enrichment was focussed on harnessing the inherent properties of Fbs1 and then enhancing its ability to effectively bind all types of N-glycans. Two approaches will be described for minimizing bias against binding complex N-glycans with terminal sialylation. First, we discovered that high salt improves wild-type (wt) Fbs1 binding to sialylated complex N-glycans, and second, we identified several interesting Fbs1 variant proteins that possess increased affinity to complex N-glycopeptides and N-glycoproteins. The most useful Fbs1 variant, named ‘GYR', was employed to isolate N-glycopeptides from a defined complex sample and this variant clearly outperformed wt Fbs1 with respect to unbiased capture of sialylated glycopeptides relative to high-mannose-containing glycopeptides. Finally, we applied Fbs1 GYR to enrich N-glycopeptides from trypsinized human serum proteins using an N-glyco-FASP method. After enrichment, 66% of the peptides in the Fbs1 GYR enrichment sample were identified as N-glycopeptides. Furthermore, the N-glycan profiles of the enriched and pre-enrichment samples were remarkably similar, which indicates Fbs1 GYR captured most of the N-glycopeptides. This substantially unbiased and highly efficient N-glycopeptide enrichment method has enabled the simultaneous determination of N-glycan composition and N-glycosites. We also found that Fbs1 GYR enrichment outperformed the established lectin enrichment method and offered a deeper analysis and greater coverage of the human serum N-glycoproteome.

## Results

### Fbs1 binds to diverse types of N-glycomolecules

**Figure 1:**
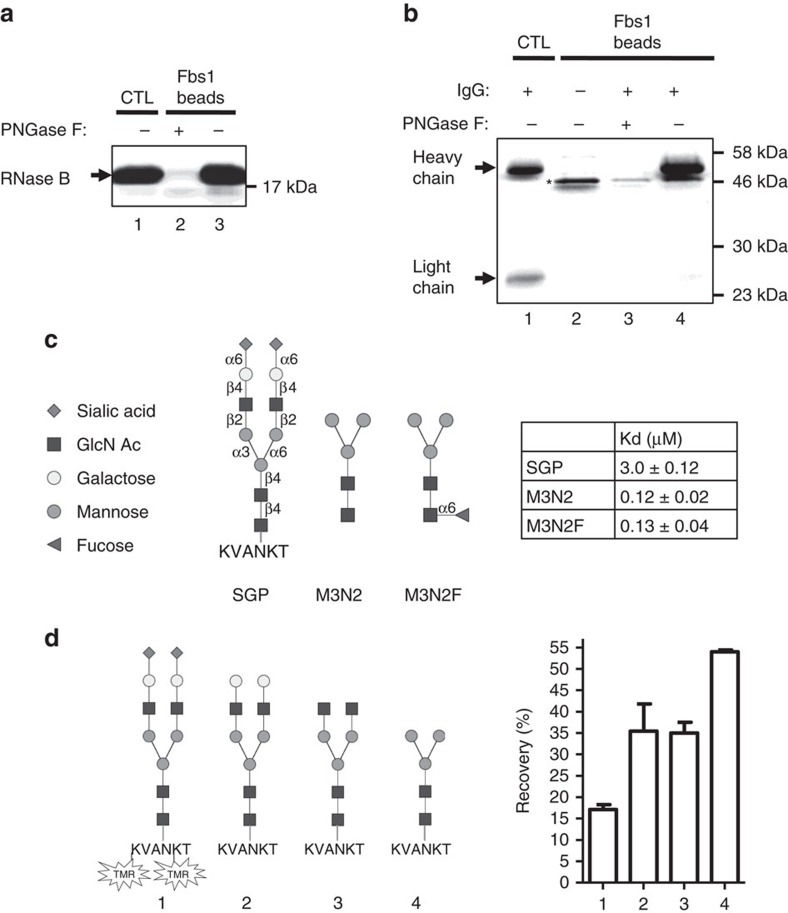
Fbs1 binds to diverse types of N-glycomolecules. (**a**) Fbs1 binding to RNase B is N-glycan dependent. RNase B or deglycosylated RNase B was subjected to an Fbs1 pulldown assay and analysed by SDS–PAGE. Lane 1, RNase B input control (CTL). Lane 2, essentially no RNase B deglycosylated by PNGase F is pulled down by Fbs1. Lane 3, RNase B with N-glycans is efficiently pulled down by Fbs1 beads. A representative SDS–PAGE gel is shown from two experiments. (**b**) Fbs1 binds to the N-glycosylated heavy chain of human IgG. Denatured and reduced human IgG were subjected to an Fbs1 pulldown assay and analysed by SDS–PAGE. Heavy chains of human IgGs are typically N-glycosylated. Lane 1 is a control showing the IgG light chain and heavy chain. Lane 2 is Fbs1 beads only. Some SNAP-Fbs1 protein leaches from the prototype Fbs1 beads (denoted by an asterisk). Lanes 3 and 4 show that only the glycosylated heavy chain is bound by Fbs1 beads. A representative SDS–PAGE gel is shown from two experiments. (**c**) Fbs1 binding affinity (Kd value) to sialylglycopeptide (SGP), M3N2 and M3N2F was measured by isothermal titration calorimetry. Structures of SGP, M3N2 and M3N2F are shown in the left panel. M3N2F is M3N2 with α1-6 fucosylation at the reducing end GlcNAc. The left panel summarizes the Kd values of SGP (*n*=4), M3N2 (*n*=5) and M3N2F (*n*=5) interacting with wt Fbs1. There is no significant difference between the Kd values of M3N2 and M3N2F (*P* value 0.85>0.05, mean±s.e.m., *t*-test, two-tailed). (**d**) wt Fbs1 shows binding bias to different N-glycopeptides. SGP was labelled with TMR fluorophore to facilitate detection. N-glycans of SGP-TMR (1) were then trimmed with exoglycosidases to produce asialo-SGP-TMR (2), SGP-TMR without sialic acids and galactose (3) and SGP-TMR without sialic acids, galactose and GlcNAc (4). Binding of the trimmed glycopeptides to Fbs1 beads was analysed. The relative binding affinity to wt Fbs1 is reported as the recovery percentage (TMR fluorescence on beads/input TMR fluorescence). For simplicity, TMR is only indicated in N-glycopeptide structure 1. Results represent the mean±s.e.m. of three replicates.

Fbs1 specifically binds to the common pentasaccharide core motif (M3N2) of N-glycans^31^,^34^. With an aim of developing an N-linked glycopeptide enrichment method, we examined the ability of wt Fbs1 to bind to a variety of glycomolecules. The human Fbs1 sugar-binding domain (residues 92–296, hereafter referred as Fbs1) was expressed as a fusion to the C-terminus of the SNAP tag to facilitate its immobilization to beads^35^. The SNAP tag protein is a mutated human DNA repair enzyme with an active site cysteine that forms a covalent linkage to benzyl guanine functional groups presented on agarose beads (BG beads). The SNAP-Fbs1 fusion protein was expressed in NEB Express *Escherichia coli* cells and highly efficient immobilization was accomplished by incubating purified SNAP-Fbs1 proteins with BG beads (see Methods section and Supplementary Fig. 1). This preparation of immobilized SNAP-Fbs1 is referred to as ‘Fbs1 beads'. In Fig. 1a, the affinity of Fbs1 for high-mannose N-glycans is shown via a pulldown assay using Fbs1 beads to capture RNase B, a protein that contains a single site modified by a variety of high-mannose N-glycans (Fig. 1a, Lane 3). Lane 2 shows that binding to RNase B is entirely dependent on the presence of N-glycan since PNGase F treatment removes the N-glycans and abolishes binding. In addition to high-mannose N-glycoproteins, we demonstrate that Fbs1 also binds to glycoproteins with complex N-glycans. Figure 1b shows that human IgG-containing complex N-glycans is also efficiently captured using Fbs1 beads. Importantly, only the N-glycosylated heavy chain is bound (Fig. 1b, Lane 4) and treatment with PNGase F indicates that binding is N-glycan dependent (Fig. 1b, Lane 3).

The interaction of Fbs1 with N-glycopeptides and free N-glycans was evaluated by isothermal calorimetric measurement (ITC). Figure 1c illustrates that Fbs1 is capable of binding to SGP, a naturally occurring glycopeptide consisting of a six amino acid peptide (KVANKT) and a bi-antennary complex N-glycan with terminal sialic acid residues (structure shown in Fig. 1c, left panel). The affinity of Fbs1 for SGP is 3.0±0.12 μM, a measurement consistent with the findings of Hagihara *et al*.^34^ who analysed Fbs1 binding to a similar sialylated substrate and arrived at a Ka=0.24 × 10^−5^ M^−1^ (equivalent to Kd=4.2 μM). These two independent analyses confirm that the presence of sialic acid negatively affects Fbs1 binding relative to high-mannose substrates where Kd measurements range from 0.24 to 0.30 μM^34^. Thus wt Fbs1 has an inherent bias against binding sialylated glycans.

In mammals, N-glycan structures may also include the heterogeneous presence of α1,6-linked fucose on the innermost GlcNAc residue of the N-glycan pentasaccharide core. ITC experiments using defined glycan substrates M3N2 and M3N2F (differing only in the presence or absence of core α1,6-linked fucose) (Fig. 1c, left panel) revealed that Fbs1 is able to bind to both substrates with equal affinity (Kd: 0.12 versus 0.13 μM, respectively; Fig. 1c). Therefore, α1-6 fucosylation of the N-glycan pentasaccharide core has no effect on Fbs1 binding.

To investigate the basis for the different Kd values for SGP versus M3N2, we performed an exoglycosidase trimming experiment. First, the lysine side chains of SGP were fluorescently labelled with tetramethylrhodamine (designated as SGP-TMR) to permit measurement of the relative concentrations of bound versus unbound substrate. Then the glycan residues in SGP-TMR (sialic acid, galactose and GlcNAc) were sequentially removed from the non-reducing end to produce a diverse set of N-glycopeptides (Fig. 1d, glycopeptides 1–4). Removal of sialic acid clearly improves recovery of the derivative glycopeptide. Furthermore, trimming to create a pentasaccharide core glycan resulted in at least a threefold improvement in recovery, a finding that is consistent with the significant difference in observed Kd values (Fig. 1d, right panel).

In typical N-glycan profiling studies, N-glycans are removed from a glycoprotein by PNGase F digestion after which liberated N-glycans are labelled with a fluorophore at their reducing end. The most commonly used fluorophore for such labelling is 2-aminobenzamide (2-AB). Thus we assessed whether the presence of 2-AB has an effect on N-glycan binding to Fbs1 beads. Supplementary Figure 2 shows that Fbs1 does not bind to M3N2-2-AB as the fluorescent signal associated with SNAP-Fbs1 beads is very low and similar to the signal associated with SNAP control beads. We reason that Fbs1 recognition of the terminal GlcNAc is abolished due to opening of the carbohydrate ring that occurs upon 2-AB labelling (Supplementary Fig. 2, structure b). However, this feature does not affect the application of Fbs1 for glycopeptide analysis since the enrichment step would be upstream of the optional 2-AB labelling step.

Considered together, these specificity studies demonstrate that Fbs1 is able to bind to both high-mannose and complex N-glycans regardless of core fucosylation. However, there is a significant reduction in binding of fully sialylated N-glycans, possibly due to their charge.

### Salt increases wt Fbs1 binding to complex N-glycans

**Figure 2:**
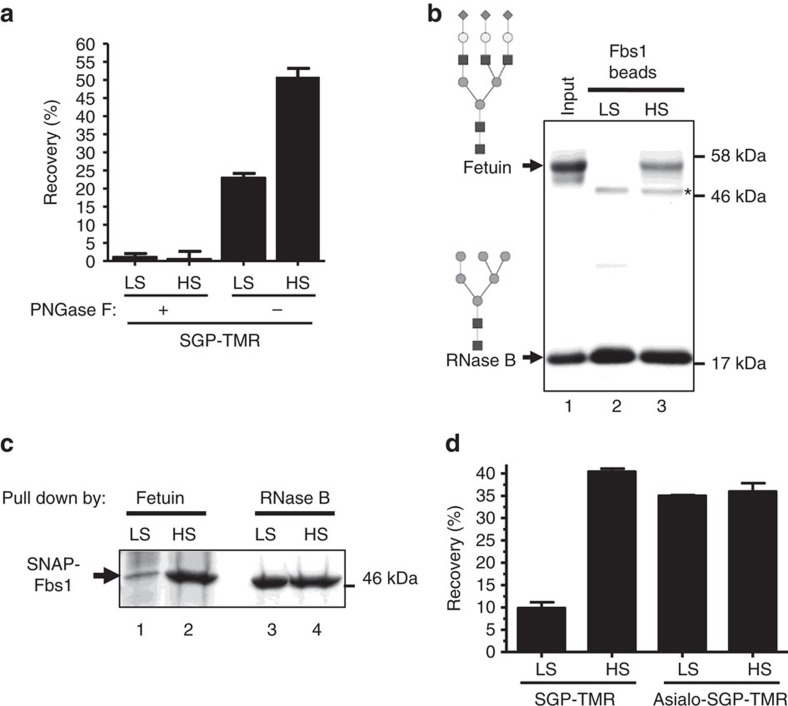
High-salt conditions increase complex N-glycomolecule binding to wt Fbs1. (**a**) The presence of 2 M NaCl increases SGP-TMR binding to wt Fbs1 in an N-glycan-dependent manner. PNGase F (+) indicates SGP-TMR was pretreated with PNGase F to cleave the glycan from the fluorophore-labelled peptide (sequence KVANKT). SGP-TMR with or without PNGase F treatment was incubated with Fbs1 beads in low-salt (LS) conditions or high-salt (HS) conditions. SGP-TMR binding to Fbs1 beads was measured, and affinity to Fbs1 is indicated by percentage of recovery (amount of bound SGP-TMR/amount of input SGP-TMR). Results represent the mean±s.e.m. of three replicates. (**b**) HS conditions increase Fbs1 binding to sialylated fetuin relative to RNase B, which contains high-mannose N-glycans. A mixture of denatured fetuin and RNase B was subjected to an Fbs1 bead pulldown assay. Lane 1 indicates the input ratio of fetuin to RNase B. Lanes 2 and 3 show the amounts of fetuin and RNase B pulled down by Fbs1 beads in LS and HS conditions. Asterisk denotes a small amount of SNAP-Fbs1 that leaches from the Fbs1 beads. N-glycan structures present within fetuin and RNase B are illustrated. A representative SDS–PAGE gel is shown from two experiments. (**c**) Reciprocal pulldown of SNAP-Fbs1 by denatured fetuin or RNase B beads in LS or HS conditions. A representative SDS–PAGE gel is shown from two experiments. (**d**) HS conditions have no effect on Fbs1 binding to asialo-SGP-TMR. SGP-TMR was trimmed with α2-3,6,8 Neuraminidase to produce asialo-SGP-TMR (structures shown in Fig. 1d, glycopeptide 1 and 2). SGP-TMR and asialo-SGP-TMR were incubated with Fbs1 beads in LS buffer or HS buffer. SGP-TMR or asialo-SGP-TMR relative affinity to Fbs1 is indicated by the recovery percentage. Results represent the mean±s.e.m. of three replicates.

Since sialylated glycans are less efficiently captured by wt Fbs1, we tested various buffer conditions in an attempt to improve recovery of complex N-glycans. For this experiment, we again used SGP-TMR as a substrate. Figure 2a demonstrates that high-salt buffer significantly improves the affinity of wt Fbs1 for SGP-TMR. PNGase F treatment confirms that the binding to SGP-TMR is N-glycan dependent. Binding affinity increases as the salt concentration is increased to ≥1.5 M (Supplementary Fig. 3). Figure 2b displays the results of a qualitative binding assay where equal amounts of RNase B and fetuin were each incubated with Fbs1 beads in low-salt versus high-salt buffers. RNase B contains single high-mannose N-linked glycans while fetuin contains sialylated complex N-linked glycans^32^. In low-salt buffer, wt Fbs1 beads pull down RNase B but almost no detectable amount of fetuin (Fig. 2b, Lane 2). However, in high-salt buffer, Fbs1 beads capture a significant amount of both fetuin and RNase B (Fig. 2b, Lane 3). This experiment shows that high ionic strength conditions enhance the binding of Fbs1 beads to sialylated complex N-glycans without negatively affecting the binding of high-mannose N-glycans. This finding is supported by a reciprocal experiment in which fetuin and RNase B were each immobilized to Affi-Gel beads and used to pull down soluble SNAP-Fbs1 (Fig. 2c). The data from this experiment show a marked increase in the affinity of Fbs1 for a complex N-linked glycoprotein in high-salt conditions (Fig. 2c, Lane 1 versus Lane 2). Furthermore, ITC experiments using high salt (2 M NaCl) produced a Kd value=1.4 μM±0.04 for SGP, a significant reduction from the value (3.0±0.12 μM) obtained using 50 mM NaCl.

The specific effect of ionic strength on binding sialylated N-glycans was further investigated by incubating Fbs1 beads with either SGP-TMR or SGP-TMR pretreated with α2-3,6,8 neuraminidase to remove sialic acids to create asialo-SGP-TMR. Binding was compared in both high- (2 M NaCl) and low-salt (50 mM NaCl) buffers in a pulldown assay. High ionic strength conditions significantly improved recovery of the SGP-TMR substrate while recovery of the asialo derivative was highly efficient in low- and high-salt buffer (Fig. 2d). We conclude that sialic acids presented on complex N-glycans cause a significant reduction in binding to Fbs1-beads, but this bias can be overcome using a high ionic strength buffer.

### Fbs1 mutants with high affinity to complex N-glycans

**Figure 3:**
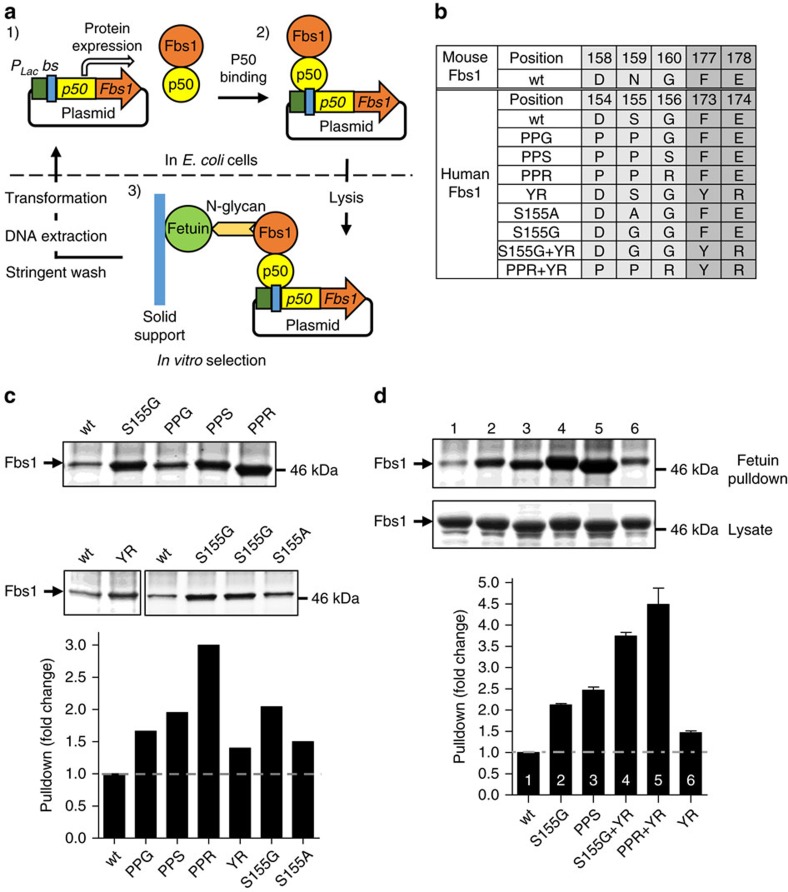
Isolation of Fbs1 mutants with improved binding affinity for complex N-glycans. (**a**) Schematic illustration of p50-Fbs1 plasmid display. (1) p50-Fbs1 fusion protein is expressed in the *E. coli* cytosol (driven by the *lac* promoter (P_*Lac*_)). The encoding plasmid contains p50 binding sites (bs). (2) p50-Fbs1 protein stably binds to the encoding plasmid via the tight interaction between p50 and its binding sites. (3) Upon gentle cell lysis, the p50-Fbs1-encoding plasmid complex is selected by the interaction between Fbs1 and complex N-glycan of fetuin (immobilized on a solid support, Affi-Gel 15). After stringent washing, Fbs1 variants with high affinity to complex N-glycans are enriched. The plasmid DNA is extracted and transformed into *E. coli* cells for the next selection cycle. (**b**) The table lists the mutants of human Fbs1 that were obtained from plasmid display screening and alanine scanning. Top two rows: the amino acid residues within mouse Fbs1 that correlate to human Fbs1. The amino acid (a. a.) position is numbered according to the full-length sequence of each respective protein. PPG, PPS, PPR and YR mutants were obtained by plasmid display screen. S155A was obtained by alanine scanning, and S155G was a further mutation based on S155A. (**c**) Relative affinity of Fbs1 variants to complex N-glycans as determined by a fetuin bead pulldown assay. *E. coli* cell lysate containing the same amount of wt Fbs1 or variant Fbs1 fusion protein was subjected to a fetuin bead pulldown assay. The bound Fbs1 protein was analysed by SDS–PAGE (upper panels) and quantified by ImageJ. The amount of bound variant Fbs1 protein was standardized to that of bound wt Fbs1 and the fold change was calculated (bottom panels, bar graphs). The relative affinity to fetuin of an Fbs1 mutant is indicated by the fold change. A representative SDS–PAGE gel is shown from two experiments. (**d**) Combination of the mutations in **c** results in Fbs1 variants with even higher affinity to complex N-glycans. All assays are the same as in **c**. A representative SDS–PAGE gel is shown from two experiments.

In addition to using high salt to overcome the inherent bias of Fbs1 against binding complex N-glycans, we sought to identify Fbs1 variants with unbiased N-glycan binding under low ionic strength conditions. A high-throughput selection method was established to isolate Fbs1 mutants with higher affinity to complex N-glycans. The method, outlined in Fig. 3a, was modelled after the plasmid display approach designed by Speight *et al*.^36^. In this method, the Fbs1 sugar-binding domain was fused to nuclear factor (NF)-κb p50 protein, a transcription factor with especially high affinity for a 10-bp sequence (5′-GGGAATTCCC-3′) strategically located at two sites within the expression plasmid (Supplementary Fig. 4a). Accordingly, expressed p50-Fbs1 fusion proteins will stably bind to the 5′-GGGAATTCCC-3′ sequences and physically link the Fbs1 variants to the encoding plasmid DNA (shown in a gel mobility shift assay in Supplementary Fig. 4b). Supplementary Figure 4c shows the results of a pilot experiment where the complexes of p50-wt Fbs1 and the encoding plasmid DNA were incubated with either immobilized RNase B or fetuin. Much higher levels of plasmid were captured with RNase B beads, indicating that the plasmid display system can differentiate between high- and low-affinity Fbs1–glycoprotein interactions.

Five amino acid clusters (Supplementary Fig. 5) within the glycan interaction surface of Fbs1 were subjected to saturation mutagenesis using randomized oligonucleotides. The mutated amino acids were chosen by analysis of the X-ray structures of mouse Fbs1 to identify amino acid substitutions that may affect recognition of the N-glycan pentasaccharide core. The five individual mutagenesis libraries were created within the context of a plasmid encoding a p50-Fbs1 fusion protein and then mixed in equal amounts (combined library complexity >1 × 10^6^) before performing plasmid display selection to isolate binding-competent Fbs1 variants. The plasmid library was used to transform *E. coli* NEB5-alpha cells that were grown to allow for expression of the p50-Fbs1 variants. After gentle lysis of the host cells, the displayed Fbs1 variants were incubated with fetuin beads. After extensive washing, the DNA–protein complexes were eluted from the fetuin beads, and the isolated plasmid was again used to transform *E. coli* cells. This enrichment procedure for high-affinity Fbs1 variants was repeated for a total of five cycles. The plasmid display results (summarized in Supplementary Fig. 5c) provided valuable information regarding amino acid substitutions that have potential to improve Fbs1 affinity to complex N-glycans. In fact, when the most commonly identified substitutions (Fig. 3b) were transferred to SNAP-Fbs1, pulldown assays reinforced the findings of the plasmid display selection process. In Fig. 3c, variants PPG, PPS, PPR (at positions 154–156) and the YR variant (F173Y/E174R) all outperformed wt Fbs1 for fetuin bead pulldown.

In addition, we performed site-directed alanine scanning to identify individual side chains that may directly influence glycan binding. Mizushima *et al*.^37^,^38^ previously reported that Man(IV) of RNase B forms a hydrogen bond with the sidechain Nδ atom of Asn-159 of mouse Fbs1. Thus we mutated the equivalent residue in human Fbs1 (Ser-155) and expected a negative impact on glycan-binding affinity. Strikingly, the S155A mutant displayed a 1.5-fold increase in pulldown of fetuin (Fig. 3c). Subsequently, position S155 was substituted with glycine (S155G) and this variant displayed a twofold increase in fetuin recovery (Fig. 3c,d). Thus the hydrogen-bonding pattern observed by X-ray crystallography does not appear critical for complex N-linked glycan binding (the affinity of S155G for RNase B was not affected, Supplementary Fig. 6). Other residues predicted to form direct contact to the RNase B glycan were mutated to alanine (human Asp154, Asp212 and Lys280) and two of the substitutions negatively affected substrate binding (Supplementary Fig. 7).

Combinatorial mutants were created using information gained from the plasmid display selection as well as the site-directed mutagenesis studies (see Fig. 3b). Overall, the PPRYR (combination of PPR and YR) and GYR (combination of S155G, F173Y and E174R) variants displayed the greatest affinity to fetuin beads (pulldown amount was at least 3.5-fold greater than wt Fbs1; Fig. 3d, Lanes 4 and 5).

### High affinity Fbs1 variants bind diverse types of N-glycans

**Figure 4:**
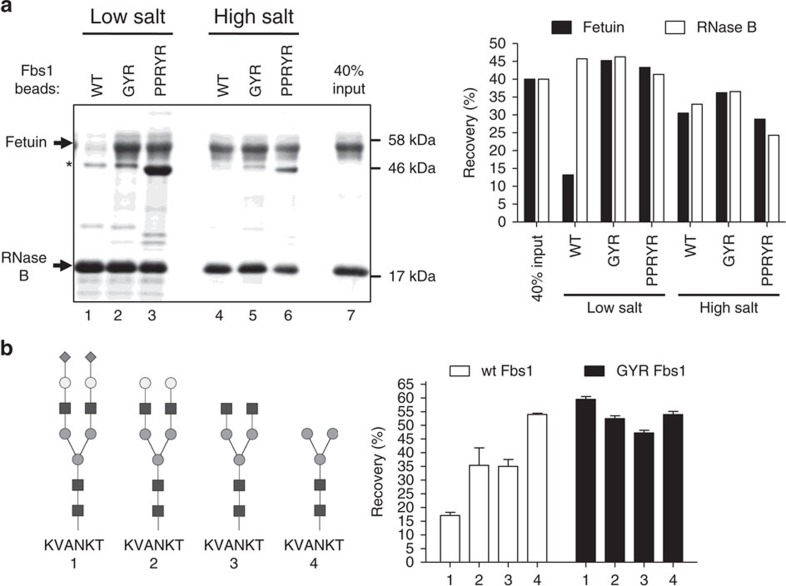
Fbs1 GYR and PPRYR variants display reduced binding bias between high-mannose and complex N-glycans. (**a**) Comparison of N-glycoprotein pulldown by wt Fbs1, Fbs1 GYR and PPRYR variant proteins. A mixture of denatured RNase B and fetuin was subjected to an Fbs1 pulldown assay with wt, GYR and PPRYR Fbs1 beads in low salt (50 mM ammonium acetate, pH7.5) and high salt (2M ammonium acetate, pH7.5). All three Fbs1 bead types were conjugated with the same amount of the respective Fbs1 protein (Supplementary Fig. 1). Left panel is the SDS–PAGE gel showing the bound (Lanes 1–6) and input ratio (Lane 7) of RNase B and fetuin. An asterisk denotes the SNAP-Fbs1 protein leaching from the Fbs1 beads. Right panel shows the recovery percentage (bound protein amount/input protein amount) of each substrate glycoprotein using the different conditions. A representative SDS–PAGE gel is shown from two experiments. (**b**) Fbs1 GYR variant binding to a diverse set of N-glycopeptides is substantially unbiased. The experiment in Fig. 1d was repeated using Fbs1 GYR beads. The data shown in Fig. 1d are presented in this figure to facilitate the comparison between wt Fbs1 and Fbs1 GYR. N-glycans of SGP-TMR (1) were trimmed with different combinations of exoglycosidases to produce asialo-SGP-TMR (2), SGP-TMR without sialic acids and galactose (3) and SGP-TMR without sialic acids, galactose and GlcNAc (4). Identities of the trimmed SGP-TMR derivatives were confirmed by LC-MS. The trimmed glycopeptides were then added to binding assays with wt Fbs1 or Fbs1 GYR beads in 50 mM ammonium acetate pH7.5. The relative binding affinity to wt Fbs1 or Fbs1 GYR is reported as the recovery percentage (TMR fluorescence on beads/input TMR fluorescence). For simplicity, TMR is not shown in the N-glycopeptide structures (1–4). Results represent the mean±s.e.m. of three replicates.

The best performing Fbs1 variants were further investigated for glycan-binding specificity. First, the fetuin:RNase B competition binding assay described in Fig. 2b was repeated using equal amounts of wt Fbs1, Fbs1 GYR (henceforth, GYR) and Fbs1 PPRYR (henceforth, PPRYR) beads (Supplementary Fig. 1) in both low- and high-salt buffers. Figure 4a shows SDS–polyacrylamide gel electrophoresis (SDS–PAGE) results where, remarkably, the GYR and PPRYR variants display no apparent bias for binding to fetuin or RNase B in low-salt buffer (Lanes 2 and 3, compared to Lane 7, in Fig. 4a). The PPRYR variant shows a slight reduction in binding to RNase B (Supplementary Fig. 6) and the PPRYR variant is also prone to aggregation during cellular overexpression and conjugation to the BG beads. For example, the asterisk in Fig. 4a indicates significant leaching of the PPRYR mutant from the beads, demonstrating that not all of the bead-associated material is covalently linked. In comparison, the GYR mutant behaved better during expression and conjugation to beads. Thus the GYR mutant was selected for further characterization.

Figure 4b further demonstrates the superior performance of the GYR variant. The GYR variant was tested for binding to SGP-TMR and three glycopeptide derivatives created by enzymatic trimming. The data from Fig. 1d were reproduced within Fig. 4b to directly compare the binding profiles of wt Fbs1 versus the GYR variant. The affinity of the GYR variant for all four glycopeptide species is very similar (recovery percentage ranged from 47% to 60%, Fig. 4b, black columns), whereas wt Fbs1 displays an obvious bias in capture of the different glycopeptides (Fig. 1d or Fig. 4b, white columns). Importantly, the GYR variant enables highly efficient N-glycopeptide recovery and binding to diverse types of N-glycans is substantially unbiased.

### N-glycopeptide enrichment by wt Fbs1 and the GYR variant

**Figure 5:**
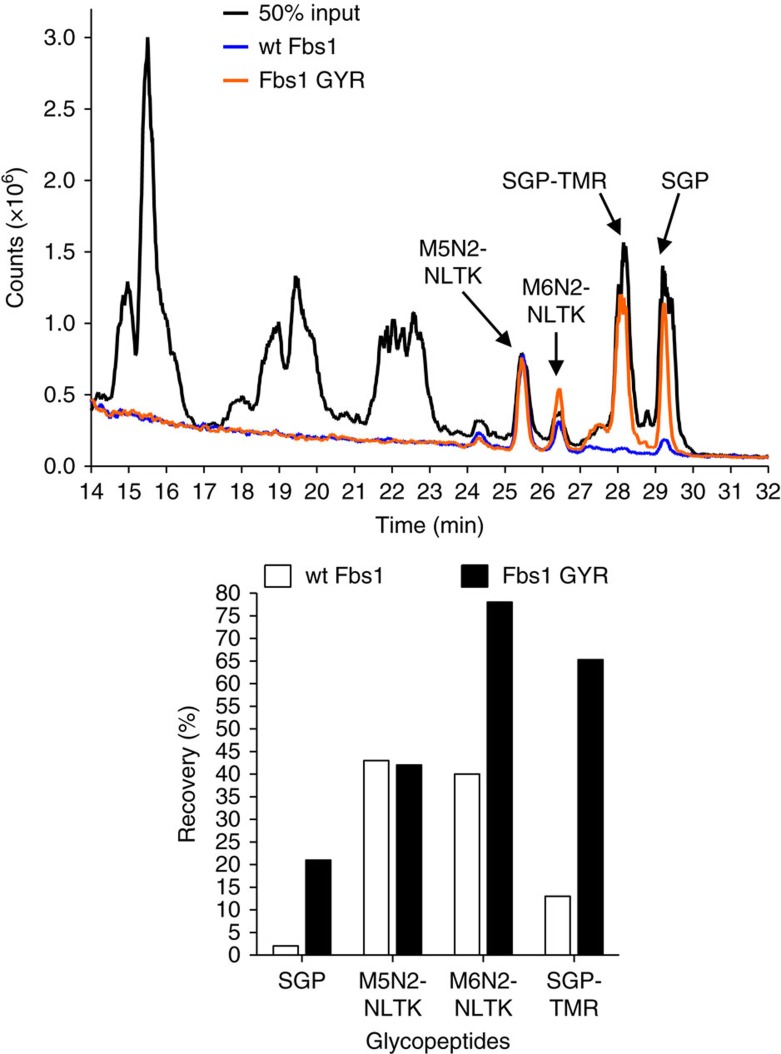
The Fbs1 GYR variant substantially improves N-glycopeptide enrichment. The total ion chromatogram (TIC) (upper panel) from an LC-MS analysis shows wt Fbs1 or Fbs1 GYR-mediated binding and enrichment of N-glycopeptides from a complex peptide mixture. The complex peptide mixture is a tryptic digest of RNase B spiked with SGP and SGP-TMR. RNaseB contains non-glycosylated peptides and two major high-mannose N-glycopeptides labelled in the enlargement as: M5N2-NLTK and M6N2-NLTK. M5N2 or M6N2 indicates 5 or 6 mannose residues and 2 GlcNAc residues, respectively. NLTK is the peptide sequence of the N-glycopeptide produced by trypsin treatment of RNase B. The enrichment was performed in low salt (50 mM ammonium acetate, pH7.5). The black line indicates the chromatogram of a 50% input mixture. The orange and blue lines indicate the chromatograms of Fbs1 GYR and wt Fbs1 enrichment samples. The major N-glycopeptide peaks (M5N2-NLTK, M6N2-NLTK, SGP and SGP-TMR) are indicated. N-glycopeptides were quantified from the extracted ion chromatogram of the LC-MS analysis. The ions with the correct monoisotopic *m/z* values, that is, M5N2-NLTK:1691.98^1-^ (theoretical, 1691.72^1-^), M6N2-NLTK: 1854.07^1-^(theoretical,1853.72^1-^) and SGP: 1433.47^2-^ (theoretical, 1433.10^2-^) were extracted, integrated and quantified. The amount of SGP-TMR was determined by fluorescence measurement of the LC elution. Recovery of each N-glycopeptide (enriched peptide amount/input peptide amount) is shown as a bar graph (lower panel). A representative TIC profile is shown from three experiments.

The selectivity of wt Fbs1 and the GYR mutant was tested by isolating and identifying the N-linked glycopeptides from a defined ‘complex' sample. RNase B tryptic peptides were generated and spiked with SGP and SGP-TMR to simulate a complex sample. Trypsin-treated RNaseB contains non-glycosylated peptides and multiple species of high-mannose N-linked glycopeptides (major species labelled as: M5N2-NLTK and M6N2-NLTK). The desired result was equivalent enrichment of each type of N-glycopeptide (sialylated versus high mannose) without contamination with non-glycosylated peptides. Figure 5 shows a total ion chromatograph of the liquid chromatography (LC)-MS-based analysis. The black line is the chromatogram of the input mixture without enrichment. The orange line is the chromatogram of the Fbs1 GYR enrichment sample and the blue line is the chromatogram of the wt Fbs1 enrichment sample. The glycomolecules elute between 24 and 30 min, whereas the non-glycosylated peptides elute before 24 min. Importantly, non-glycosylated peptides are not enriched by either wt Fbs1 or the GYR variant. The extracted ion chromatogram data (shown as a bar graph, Fig. 5) also shows that the GYR variant performs well for capturing high-mannose-containing glycopeptides M5N2-NLTK and M6N2-NLTK. Most importantly, enrichment of the sialylated glycan species is significantly improved by use of the GYR variant.

### Application of Fbs1 GYR to identify N-glycosites in serum

**Figure 6:**
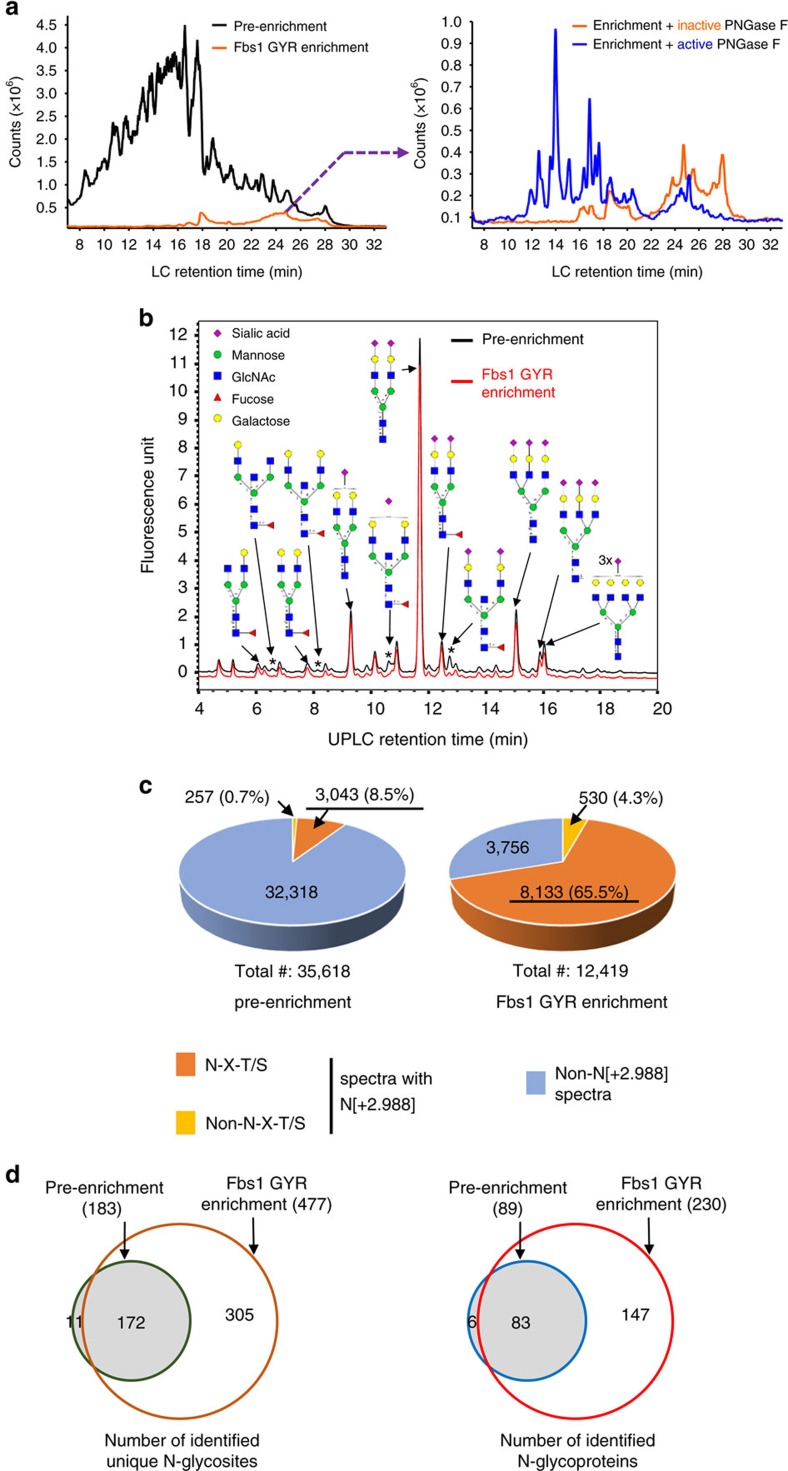
N-glycopeptide enrichment from human serum tryptic peptides by Fbs1 GYR. (**a**) Left panel: The total ion chromatogram (TIC) from an LC-MS analysis of HSA-depleted human serum tryptic peptides without Fbs1 enrichment (pre-enrichment) or the peptides from Fbs1 GYR enrichment. Right panel: TIC from an LC-MS analysis of peptides enriched by Fbs1 GYR treated with active PNGase F (blue curve) or heat-inactivated PNGase F (95 °C, 10 min) (orange curve). A representative TIC profile is shown from three experiments. (**b**) N-glycan profiling of human serum (-HSA) tryptic peptides without Fbs1 enrichment (pre-enrichment, dark curve) or the peptides from Fbs1 GYR Enrichment (red curve) by 2-AB labelling and UPLC. Glycan structures are assigned to major peaks according to the glucose unit of each peak (Supplementary Fig. 8). The reduced peaks in the Fbs1 GYR enrichment sample are labelled with an ‘*' The red curve is offset for better comparison with the black curve. A representative glycan profile is shown from two experiments. (**c**) N-glycosite identification using PNGase F deglycosylation in ^18^O water. HSA-depleted human serum tryptic peptides without Fbs1 enrichment (pre-enrichment) or enriched by Fbs1 GYR (Fbs1 GYR enrichment) were deglycosylated by PNGase F in ^18^O water. Upon N-glycan removal, asparagine (N) in the N-glycosite is deamidated to aspartic acid resulting in a peptide tagged with an additional 2.988 daltons. Peptide spectra with N[+2.988] within the canonical N-glycosylation motif N-X-T/S can be confidently assigned as N-glycopeptides. Detailed MS spectrum information is in Supplementary Data 1 and 2. The MS data were combined from two mass spectrometric experiments. (**d**) Comparison of unique N-glycosites and N-glycoproteins identified without (pre-enrichment) or with Fbs1 GYR enrichment. Left panel: 183 and 477 unique N-glycosites were identified in pre-enrichment and Fbs1 GYR enrichment, respectively. One hundred and seventy-two (94%) of the 183 N-glycosites in the pre-enrichment were also identified in the Fbs1 GYR enrichment sample. Right panel: 89 and 230 N-glycoproteins were identified in pre-enrichment and Fbs1 GYR enrichment, respectively. Eighty-three (93%) of the 89 N-glycoprotein in the pre-enrichment were also identified in the Fbs1 GYR enrichment sample. Detailed N-glycosite and N-glycoprotein information is in Supplementary Data 1 and 2.

We assessed N-glycopeptide enrichment from a biological sample using Fbs1 GYR. In this study, the N-glyco-FASP method^24^,^39^,^40^ was employed. Tryptic peptides prepared from human serum albumin (HSA)-depleted human serum without enrichment (pre-enrichment) or with Fbs1 GYR enrichment were subjected to direct LC-MS analysis (Fig. 6a), N-glycan profiling analysis (Fig. 6b) and N-glycosite identification/deglycoproteomics^10^ using the PNGase F/^18^O water method^24^,^39^ (Fig. 6c,d). Figure 6a shows the total ion chromatograph from the LC-MS analysis of pre-enrichment and Fbs1 GYR enrichment (Left panel). Approximately 14% of peptides were retained by Fbs1 GYR. When the peptides enriched by Fbs1 GYR were treated with active PNGase F to remove N-glycans, the overall peptide profile shifted to earlier retention time area in the HILIC LC due to the removal of hydrophilic N-glycans from the peptides. This suggests that a majority of the enriched peptides are N-glycopeptides. We also examined the recovery efficiency of N-glycopeptides in the Fbs1 GYR enrichment sample. We compared the 2-AB-labelled N-glycan profile from pre-enrichment and Fbs1 GYR enrichment samples. Although four small peaks (labelled with asterisk (*), corresponding to four bisecting N-glycans) were reduced in the Fbs1 GYR enrichment sample, Fig. 6b shows the two N-glycan profiles (peak pattern and peak height) are remarkably similar. These N-glycan profiles suggest Fbs1 GYR efficiently binds most N-glycans and thus recovers most of the N-glycopeptides from human serum samples.

Next we examined Fbs1 GYR enrichment in detail by N-glycosite identification or deglycoproteomics^10^ using PNGase F deglycosylation in the presence of ^18^O water, which tags the deglycosylated peptides with 2.988 daltons due to conversion of asparagine (N) at the N-glycosylation site to aspartic acid (D) and incorporation of ^18^O. The peptide spectra with isotope tagging [+2.988] at N within the canonical N-glycosylation motif N-X-T/S can be confidently assigned as an N-glycopeptide^24^,^41^. Although some N-glycosylations occur at non-N-X-T/S motifs^42^, we excluded the peptide spectra with an N[+2.988] tag at non-N-X-T/S motifs since these sites need further confirmation. Figure 6c and Supplementary Data 1 and 2 show Fbs1 GYR enrichment greatly increases the N-glycopeptide fraction in the samples (65.5% in Fbs1 GYR enrichment versus 8.5% in pre-enrichment), thus greatly reducing the sample complexity. This enabled the identification of many more unique N-glycosites. From the same amount of starting human serum tryptic peptides, 477 unique N-glycosites were identified after Fbs1 GYR enrichment, while only 183 unique N-glycosites were identified without Fbs1 GYR enrichment (pre-enrichment), of which 172 N-glycosites (94%) were also identified by Fbs1 GYR enrichment (Fig. 6d, left panel and Supplementary Data 1 and 2). The unique N-glycosites were assigned to 89 and 230 N-glycoproteins in pre-enrichment and Fbs1 GYR enrichment samples, respectively (Fig. 6d, right panel and Supplementary Data 1 and 2). Eighty-three (93%) N-glycoproteins identified in pre-enrichment samples were also identified in Fbs1 GYR enrichment samples.

### Fbs1 GYR improves intact N-glycopeptide identification

**Figure 7:**
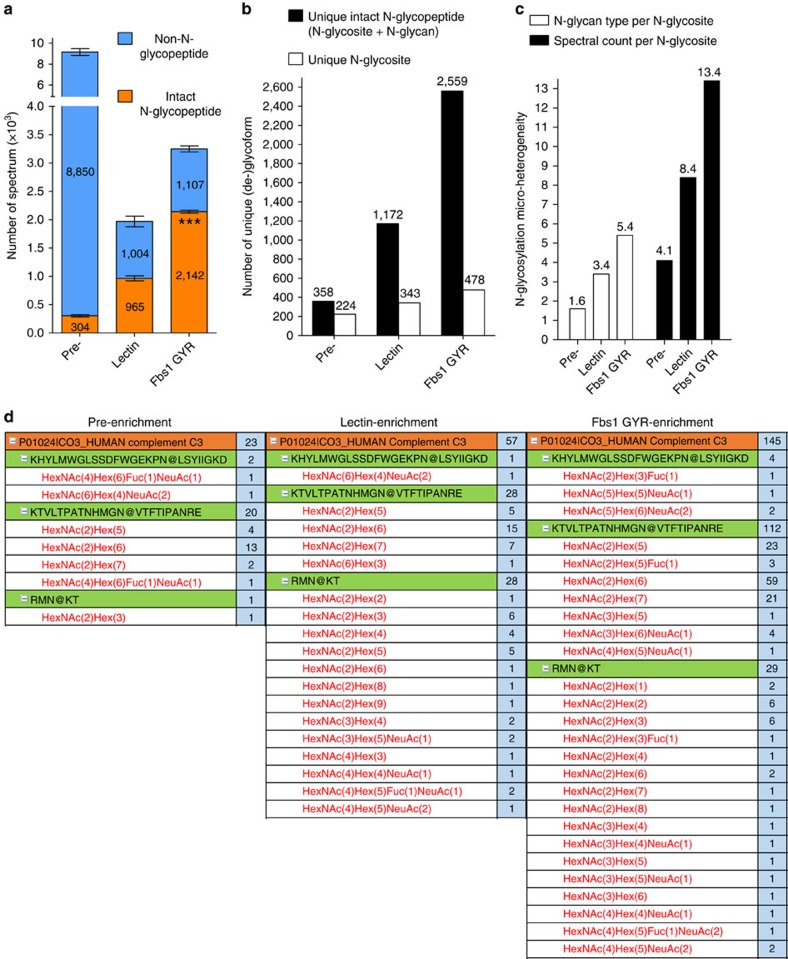
Fbs1 GYR enrichment greatly improves intact N-glycopeptide identification. (**a**) Intact N-glycopeptide identification using Fbs1 GYR enrichment, lectin enrichment or no enrichment (pre-enrichment, pre-). MS data were obtained from three mass spectrometric experiments (*n*=3). The average number of MS spectra of intact N-glycopeptides (orange) and non-N-glycopeptides (blue) are shown in the bar graph. The number of N-glycopeptide spectrum from Fbs1 GYR enrichment was significantly higher relative to lectin enrichment (***P<0.001, mean±s.e.m., *t*-test, two-tailed). Detailed intact N-glycopeptide MS data is in Supplementary Data 3–5. (**b**) Comparison of unique intact N-glycopeptides and unique N-glycosites identified from pre-enrichment, lectin enrichment and Fbs1 GYR enrichment. The numbers were generated from combining three intact N-glycopeptide MS data sets (Supplementary Data 3–5). Peptide miscleavage was manually corrected. (**c**) Comparison of N-glycosylation micro-heterogeneity identified from pre-enrichment, lectin enrichment and Fbs1 GYR enrichment. The overall determination of N-glycosylation micro-heterogeneity is indexed by glycan type per glycosite (number of unique intact N-glycopeptides/number of unique N-glycosites) and spectral count per glycosite (number of spectral counts/number of unique N-glycosites). (**d**) Micro-heterogeneity of N-glycosylation in human Complement C3 protein (as an example) illustrated by intact N-glycopeptide identification without enrichment (pre-enrichment) or using Fbs1 GYR enrichment and lectin enrichment. The orange row indicates N-glycoprotein identification. Beneath protein identification, N-glycosites are listed (in green rows). Beneath N-glycosites, N-glycan compositions attached to this N-glycosite are listed (in red). N@ indicates the asparagine with N-glycan modification. The light blue columns indicate the spectral counts of each N-glycoform. A full list of N-glycosylation micro-heterogeneity illustrated by intact N-glycopeptide identification is shown in Supplementary Data 6.

We have shown above that Fbs1 GYR significantly improves N-glycosite identification (deglycoproteomics) from human serum. Each N-glycosite may contain a different population of N-glycan modifications (micro-heterogeneity), which can be predictive markers for disease states or specific biological function^10^,^13^. Standard N-glycosite identification by PNGase F treatment in ^18^O water results in the loss of information pertaining to which N-glycans are present at each glycosite. Since Fbs1 GYR enrichment maintains the linkage of each N-glycan to each respective N-glycosite, and enrichment levels are >65%, our protocol allows simultaneous identification of the N-glycan diversity at a given N-glycosite. To demonstrate this, we applied Fbs1 GYR to enrich and identify intact N-glycopeptides from IgG-depleted human serum, which contains fewer glycomolecules compared to HSA-depleted human serum. We also compared Fbs1 GYR enrichment with lectin (ConA, WGA and RCA_120_ mixture) enrichment for intact N-glycopeptide identification. Tryptic peptides prepared from IgG-depleted human serum without enrichment (pre-, or pre-enrichment), with lectin enrichment, or with Fbs1 GYR enrichment were subjected to LC-MS/MS using the higher energy collisional dissociation (HCD) fragmentation method^11^. Intact N-glycopeptides were identified by using Byonic software to compare the HCD spectra against the human proteome database (uniprot human up000005640) and the 57 most common N-glycan structures in human plasma. Most importantly, Fbs1 GYR enrichment revealed more intact N-glycopeptides relative to lectin enrichment. With the same input of tryptic peptides (1 μg peptides per MS run), an average of 2,142 N-glycopeptide spectra were identified after Fbs1 GYR enrichment, which is 2.2-fold more than the number (965) of N-glycopeptide spectra identified using lectin enrichment, and 7-fold more than the number (304) identified without enrichment (Fig. 7a and Supplementary Data 3–5). Following Fbs1 GYR enrichment 66% of total spectra were assigned to N-glycopeptides, while only 49% and 3.3% of spectra were assigned as N-glycopeptides in lectin enrichment and pre-enrichment samples, respectively (Fig. 7a). Combining all three MS runs, Fbs1 GYR enrichment enabled identification of 2,559 unique, intact N-glycopeptides, which is a 2.2-fold and 7-fold improvement over the unique peptides identified using lectin enrichment (1,172) and pre-enrichment (358), respectively (Fig. 7b). Fbs1 GYR enrichment also identified 478 unique N-glycosites, compared to 343 following lectin enrichment, and 224 unique N-glycosites in the pre-enrichment sample (Fig. 7b). Our data demonstrate that Fbs1 GYR enrichment can greatly improve identification of micro-heterogeneity of N-glycosylation, which can be indexed as the average number of glycan types per glycosite (number of unique N-glycopeptides/number of unique N-glycosites) and the average number of spectral counts per glycosite (number of spectral counts/number of unique N-glycosites). Figure 7c shows that Fbs1 GYR enrichment results in an average of 5.4 glycan types per N-glycosite and 13.4 spectral counts per N-glycosite, a 3.3-fold improvement over the values before enrichment. Additionally, lectin enrichment only provides a two-fold improvement versus pre-enrichment in the micro-heterogeneity analysis (Fig. 7c). Figure 7d shows the N-glycosylation micro-heterogeneity analysis for a single potential disease biomarker, human Complement C3^43^. In this case, Fbs1 GYR enrichment clearly allows identification of more glycan types and spectra at each glycosite relative to the pre-enrichment and lectin enrichment samples. The complete data set from the N-glycosylation micro-heterogeneity analysis is presented in Supplementary Data 6. Overall, this experiment demonstrates that Fbs1 GYR enrichment enables more effective intact N-glycopeptide analysis with greatly expanded coverage and reveals a deeper analysis of micro-heterogeneity of N-glycosylation.

## Discussion

The human glycoproteome is rapidly emerging as an attractive reservoir of potential biomarkers of both health and disease^44^,^45^. The plasma glycoproteome, in particular, is a valuable source for biomarkers so one may envisage rapid blood tests for early disease detection^46^. In order to achieve this goal, researchers are developing highly sophisticated LC/MS methods to characterize the diverse types of glycan modifications^12^,^15^,^47^,^48^,^49^,^50^. Even as the data set grows, accurate determination of glycan composition and the associated glycosylation sites remains challenging^51^. Effective enrichment procedures are critically important to permit accurate, comprehensive analysis of the glycopeptide profile of patient samples.

In the present study, we developed a novel strategy for selective isolation of N-linked glycopeptides *en masse* from complex peptide mixtures or biological samples. We made use of the glycan-binding properties of Fbs1, a eukaryotic lectin-like protein that normally functions in a ubiquitin-mediated process to eliminate misfolded N-glycosylated proteins. Fbs1 binds a Man_3_GlcNAc_2_ pentasaccharide that is common to all mammalian N-glycan species. Specifically, we show that an immobilized form of Fbs1 is able to bind to both high-mannose and complex N-glycans regardless of the presence or absence of α1,6-linked fucose attached to the innermost GlcNAc residue. In low ionic strength buffer, Fbs1 preferentially binds to high-mannose N-glycans over sialylated complex N-glycans. However, we showed two approaches to mitigate this bias. First, wt Fbs1 binding to sialylated complex N-glycans was greatly improved in a high ionic strength buffer. Second, we isolated mutant forms of Fbs1 that bind to both complex N-glycans and high-mannose N-glycans with similar efficiency. The best performing Fbs1 variant contains the following amino acid substitutions: S155G, F173Y, and E174R. At position 155, we first discovered that a smaller side chain (alanine) resulted in greater fetuin pulldown (Fig. 3c). Then further mutagenesis to glycine resulted in even higher affinity to complex N-glycans, most likely due to removal of steric hindrance. The subtle change from phenylalanine to tyrosine is more difficult to explain. We speculate that the addition of a single hydroxyl group may positively affect sugar ring binding. Finally, the E174R charge reversal substitution is theoretically important for improved recognition of negatively charged sialic acid residues.

Over the past 15 years, other important technologies have been developed for glycomolecule enrichment. Most are based on the hydrophilic properties of the sugar residues comprising both N- and O-linked modifications. Unfortunately, these methods may enrich other types of biomolecules. Methods specific for sialic acid capture also do not discriminate between N- and O-linked modifications^15^. PNGase F specifically liberates N-glycans for accurate identification and if the hydrolysis reaction is conducted in the presence of ^18^O water the site of protein attachment will be distinctly labelled (Asn to Asp-^18^O). But information pertaining to the glycan composition at that site is lost upon hydrolysis. Our objective was to develop a method for unbiased N-glycopeptide enrichment that allows for simultaneous determination of glycan composition and the protein attachment site. Rather than utilize a chemical approach, we looked to nature for a protein with desirable properties. Lectins have been employed for many years for glycomolecule characterization and/or isolation. Yet no single lectin has been exploited for selective enrichment of N-linked glycopeptides.

The inherent properties of Fbs1 are attractive and these properties were improved through a focussed protein engineering approach. Several protein display technologies were considered for the evolution of Fbs1 (ref. 52). In our hands, plasmid display worked extremely well for the identification of high-affinity-binding proteins. Five cycles of panning against immobilized fetuin were accomplished in 5 days, and the information from the selected mutants resulted in discovery of higher-binding-affinity variants of human Fbs1. Thus we recommend this method as an alternative to M13 phage display or cell-surface display technologies since not all proteins are amenable to membrane translocation or the folding environment of the secretory pathway. With plasmid display, expression of the protein library occurs in the *E. coli* cytoplasm. Cellular expression of the library proteins is combined with *in vitro* selection against an immobilized glycan substrate all the while maintaining the genotype:phenotype linkage necessary for a high-throughput method. We envisage that plasmid display will be a valuable approach for the evolution of other carbohydrate-binding proteins.

Many important biological activities are affected by protein glycosylation, including protein folding, protein metabolism, protein–protein interactions, immune cell recognition and intercellular signaling. Given the emerging interest in glycoproteins as biomarkers, a need exists for readily isolating and characterizing low-abundance glycoproteins from complex biological samples. The following features facilitate the application of the GYR variant in a simple workflow for N-glycopeptide enrichment: (1) the SNAP fusion partner enables rapid, efficient immobilization; (2) alternatively, the SNAP-Fbs1 fusion protein can also be employed in an N-glyco-FASP protocol; (3) N-glycopeptide capture may be accomplished in many buffer conditions, preferably a volatile low-salt buffer such as 50 mM ammonium acetate or ammonium bicarbonate; (4) enriched N-glycopeptides may be eluted in 50% acetonitrile or 50% formic acid and directly analysed by MS; and (5) substantially unbiased capture of intact N-glycopeptides (rather than liberated N-glycans or deglycosylated peptides) allows for simultaneous identification of N-glycan composition and the respective glycosite.

In both deglycoproteomic (Fig. 6) and glycoproteomic (Fig. 7) studies, N-glycopeptide abundance in the human serum samples was increased to 66% after Fbs1 GYR enrichment. This complexity reduction allows for more effective sample analysis during MS, which will improve large-scale glycoproteomic studies. The N-glycan profile from Fig. 6b indicates that Fbs1 GYR can capture most of the N-glycans in serum with similar efficiency. However, there is one apparent limitation for Fbs1 enrichment. N-glycan profiles shown in Fig. 6b suggest that Fbs1 GYR has a low affinity for bisecting N-glycans. According to the Fbs1 X-ray structure^37^,^38^, the presence of a single bisecting GlcNAc residue may create a steric hindrance for Fbs1 binding.

N-glycomolecule enrichment is routinely accomplished by HILIC, chemical methods or by capture with lectins. For example, the specific mixture of ConA, WGA and RCA_120_ lectins has been applied with success to enrich for N-glycan containing peptides^24^. Therefore, we directly compared Fbs1 GYR to this lectin mixture and found that the engineered, recombinant Fbs1 protein outperformed this mixture of non-recombinant proteins (Fig. 7). Alternatively, HILIC enrichment may be used for glycoproteomic studies, but it is more commonly applied for glycan profiling. Notably, HILIC enrichment is not specific for N-glycans and N-glycopeptide enrichment to only 30% has been achieved previously^53^. For intact N-glycopeptide identification, HILIC requires an additional chromatography step to achieve sufficient enrichment^18^,^19^. Hydrazide-based methods can improve N-glycopeptide abundance to 80–90% (refs 14, 53), but this either results in the loss of N-glycan information^12^,^14^ or only sialyated glycans may be captured^15^. Fbs1 enrichment improves N-glycopeptide abundance to 66% in a specific and non-destructive manner. This substantially unbiased and highly efficient N-glycopeptide enrichment method enables the simultaneous determination of N-glycan composition and N-glycosites with a deeper coverage (compared to lectin enrichment) and allows large-scale N-glycoproteomic studies due to greatly reduced sample complexity. In conclusion, we propose that the Fbs1 GYR variant may be the centrepiece of analysis methods requiring selective N-glycomolecule enrichment.

## Methods

### Materials

RNase B, fetuin, PNGase F, α2-3,6,8 Neuraminidase, β1-4 Galactosidase S, β-N-Acetylglucosaminidase S and MS-grade Trypsin were from New England Biolabs (NEB) (Ipswich, MA). Human IgG was purchased from Bethyl Laboratories, Inc. (Montgomery, TX). Glycans M3N2, M3N2F and M3N2-2-AB were purchased from Prozyme (Hayward, CA). SGP was obtained from Fushimi Pharmaceutical Co., Ltd. (Japan). SGP-TMR, SGP labelled with two TMR fluorophores on the peptide portion (via lysines), was prepared using Tetramethylrhodamine Protein Labeling Kit from Genaxxon Bioscience (Germany) according to the manufacturer's protocol. SGP-TMR was purified by HPLC (to separate non-labelled SGP and SGP-TMR) and polyacrylamide desalting columns (Thermo Fisher Scientific, Waltham, MA) to separate SGP-TMR and free TMR. Human serum, Affi-Gel Blue beads, Protein A beads and ^18^O water (^18^O, 97%) was purchased from Sigma (St Louis, MO), Bio-Rad (Hercules, CA), Thermo Fisher Scientific and Cambridge Isotope Laboratories, Inc. (Andover, MA), respectively. Lectins (ConA, WGA and RCA_120_) were purchased from Sigma.

All the constructs were made using NEBuilder HiFi DNA Assembly Cloning Kit (NEB), and gene/DNA oligonucleotide synthesis was provided by IDT (Coralville, IA). For expression of SNAP-Fbs1 in *E. coli*, wt human *Fbs1* gene encoding amino acid residues 92–296 was cloned into pSNAP-tag(T7) expression vector (NEB). The construct encodes a SNAP-Fbs1 fusion protein with SNAP tag at N-terminus and followed by Fbs1 (Supplementary Fig. 9).

### Fbs1 bead preparation

Purified wt or mutant SNAP-Fbs1 proteins were incubated with SNAP-Capture Pulldown Resin (NEB) (also called benzylguanine beads or BG beads) at 4 mg protein per 1 ml resin in binding buffer (20 mM Tris.HCl pH 7.5, 50 mM NaCl, 1 mM EDTA) supplemented with 5 mM dithiothreitol (DTT) at 4 °C overnight. The conjugated beads were washed with >40 resin bed volumes of the binding buffer and then suspended in 50 mM ammonium acetate, pH 7.5.

### Fbs1 bead pulldown assay

In order to expose N-glycans to Fbs1, N-glycoproteins were first denatured. RNase B or human IgG was denatured by boiling in 0.5% SDS and 40 mM DTT. After boiling, 1% NP-40 was added to counteract the effect of SDS. The denatured RNase B or human IgG was either untreated or treated with PNGase F (for 1 h at 37 °C in 50 mM sodium phosphate, pH 7.5) to remove N-glycans as indicated in the figures. The mixture of RNase B and fetuin was denatured by boiling for 10 min in the presence of 1 × Rapid PNGase F buffer (NEB). The denatured proteins were incubated with Fbs1 beads at 4 °C for 1 h. After incubation, the beads were washed, and the bound proteins on the beads were eluted in 1 × SDS gel loading buffer and analysed by SDS–PAGE/Coomassie blue staining. ImageJ (National Institutes of Health) was used to quantify the protein bands.

### Fetuin or RNase B bead preparation

RNase B and fetuin beads were prepared according to Yoshida *et al*.^32^,^54^. Briefly, RNase B and fetuin were conjugated to Affi-Gel 10 and Affi-Gel 15 (Bio-Rad, Hercules, CA) in 0.1 M MOPS, pH 7.5, respectively, and then RNase B or fetuin on the Affi-Gel beads was denatured by incubating with 6 M Guanidine-HCl overnight at room temperature. The beads were then washed with 20 mM Tris.HCl pH 7.5, 50 mM NaCl and 1 mM EDTA to remove Guanidine-HCl.

### Fetuin or RNase B bead pulldown assay

One millilitre of the *E. coli* cell lysate containing wt Fbs1 or Fbs1 mutant proteins was incubated with 50 μl fetuin or RNase B beads at 4 °C for 1 h in optimal binding buffers (20 mM Tris.HCl pH 7.5, 1 mM EDTA, 50 mM NaCl (for low-salt conditions) or 2 M NaCl (for high-salt conditions)) supplemented with 30% (v/v) of Pierce Protein-Free Blocking buffer (Thermo Fisher Scientific). After incubation, the beads were washed three times with 1 ml of the corresponding binding buffers. In each wash, 30-gauge insulin syringes (Becton Dickinson, Franklin Lakes, NJ) were used to remove residual buffer. SDS–PAGE loading buffer was used to elute Fbs1 bound to the beads.

### Isothermal titration calorimetry

Purified SNAP-Fbs1 protein was dialysed against dialysis buffers (20 mM Tris.HCl pH 7.5, 1 mM EDTA, 50 mM NaCl (low-salt condition) or 2 M NaCl (high-salt condition)). M3N2 or M3N2F glycans or SGP were also dissolved in the same dialysis buffer. A NanoITC instrument (TA Instruments, Lindon, UT) with 170 μl cell volume and 50 μl buret volume was used. Every 300 s, 2.97 μl of 90 μM glycan was automatically injected into 9 μM Fbs1 solution or 2.97 μl of 270 μM SGP was automatically injected into 27 μM Fbs1 solution. The data were fitted using a single site binding model (Nanoanalyze software, TA Instruments), and the Kd was calculated.

### SGP-TMR or M3N2-2-AB binding assay

SGP-TMR or M3N2-2-AB was incubated with Fbs1 beads at 4 °C for 1 h. The Fbs1 beads were then centrifuged and supernatant was collected. TMR or 2-AB fluorescence in the supernatant indicates the level of SGP-TMR or M3N2-2-AB not bound by Fbs1, respectively. TMR or 2-AB fluorescence in the supernatant was measured using a SpectraMax M5 fluorometer (excitation 555 nm and emission 595 nm with 590 nm cutoff for TMR, and excitation 320 nm and emission 425 nm with 420 nm cutoff for 2-AB). SGP-TMR or M3N2-2-AB bound to Fbs1 beads was calculated by subtracting fluorescence measured in supernatant from input fluorescence. Recovery percentage was calculated according to the amount of fluorescence on the beads divided by input fluorescence.

Binding assays (including pulldown assays) were performed in either low- or high-salt buffers. Low-salt buffer: 50 mM NaCl, 20 mM Tris.HCl, 1 mM EDTA, pH 7.5, or 50 mM ammonium acetate, pH 7.5. High-salt buffer: 2 M NaCl, 20 mM Tris.HCl, 1 mM EDTA, pH 7.5, or 2 M ammonium acetate, pH 7.5. Ammonium acetate was chosen as an alternative to sodium chloride due to its volatility and compatibility with MS.

### SGP-TMR N-glycan trimming

SGP-TMR was incubated for 2 h at 37 °C with α2-3,6,8 Neuraminidase to remove the sialic acids; or with α2-3,6,8 Neuraminidase and β1-4 Galactosidase S together to remove sialic acids and galactose, or with α2-3,6,8 Neuraminidase, β1-4 Galactosidase S and β-N-Acetylglucosaminidase S together to remove sialic acids, galactose and GlcNAc according to New England Biolabs protocols. The trimmed glycopeptides were then directly used in the Fbs1 bead-binding assay as described above.

### Plasmid display constructs

Plasmid display constructs were designed according to the method described by Speight *et al*.^36^. The backbone of the plasmid display constructs is pUC19 with the ampicillin resistance gene replaced by kanamycin resistance gene (termed as pUCKan). Two NF-κb p50 DNA-binding sequences, 5′-GGGAATTCCC-3′, were inserted 36 bp upstream of the CAP protein-binding site and Lac operator, respectively. The Fbs1 plasmid display construct (termed as pUCKan-p50-Fbs1) contains a fusion of the human *Fbs1* gene to the 3′ end of the human NF-κb p50 gene. The fusion gene is under control of *lac* promoter (P_*lac*_). The p50 gene encodes the DNA-binding domain of NF-κb p50 protein (amino acid residues 40–366), and the human *Fbs1* gene encodes the Fbs1 sugar-binding domain (amino acids residues 92–296). These two domains are connected by a linker sequence, GTSAAAGSGS. The control plasmid (pUCKan-p50) for plasmid display is pUCKan-p50 (*Fbs1* gene is deleted).

### Fbs1 saturation mutagenesis library for plasmid display

Five clusters containing 2–3 adjacent amino acids from the predicted Fbs1: N-glycan interaction surface were chosen for saturation mutagenesis. The mutagenesis oligonucleotide sequences are available upon request. The mutagenesis reactions were performed on pUCKan-p50-Fbs1 using Q5 Site-Directed Mutagenesis Kit (NEB). Mutagenesis of each cluster created a sublibrary, and the five sublibraries were mixed in equal amounts to create a final library for plasmid display screening.

### Plasmid display selection

Plasmid display was performed according to the protocol described by Speight *et al*.^36^. Briefly, NEB 5-alpha *E. coli* cells (NEB) harbouring p50-Fbs1 constructs were grown overnight without inducer. The cells were gently lysed using a spheroplast method: *E. coli* cells were converted to spheroplasts using 100 mM Tris.HCl pH 8.0, 20% sucrose, and 0.25 mg ml^−1^ lysozyme. The spheroplasts were lysed in 10 ml KBD buffer (10 mM Tris.HCl pH 7.4, 50 mM potassium glutamate, 3 mM DTT, 0.02% v/v Triton x-100, 10% glycerol, 0.1 mg ml^−1^ sonicated herring sperm DNA (Sigma). After clearing the cell debris by centrifugation at 30,000*g* for 15 min, the cell lysate was incubated with 0.5 ml fetuin beads at 4 °C for 1 h. The beads were then washed with 9 × 10 ml KBD buffer. In all, 0.5 ml P2 buffer from the Qiagen QIAprep kit was used to elute the bound plasmid-p50-Fbs1 complex. The plasmid DNA was then purified from the eluent according to the protocol of the Qiagen QIAprep Kit. The purified plasmid DNA was electroporated into NEB 5-alpha electrocompetent *E. coli* cells (NEB) for the next cycle of selection.

### Preparation of tryptic peptides from human serum

HSA-depleted or IgG-depleted human serum was prepared by incubating human serum with Affi-Gel Blue beads (Bio-Rad) or Protein A beads (Thermo Fisher Scientific) according to the manufacturer's protocols, respectively. Tryptic peptides of human serum were prepared according to the filter-aided sample preparation (FASP) protocol as described^24^,^40^. Briefly, 300–400 μg protein (5–10 μl serum) is mixed with 100 μl EB buffer (4% SDS, 100 mM DTT in 100 mM Tris.HCl pH 7.5) and then denatured at 95 °C for 3 min. The sample buffer is then exchanged to UA buffer (fresh 8 M urea in 0.1 M Tris.HCl pH 8.5) in a filter unit (Ultracel YM-30, Millipore, Germany) by mixing twice with 200 μl of UA buffer and centrifuging at 14,000*g* for 15 min to remove SDS and DTT. The serum protein in UA buffer is then mixed with 100 μl IAA solution (fresh 0.05 M iodoacetamide in UA buffer) and incubated for 20 min in the dark. The non-reacted IAA is removed by centrifugation at 14,000*g* for 10 min and addition of new UA buffer (100 μl) three times. The sample buffer is then exchanged to 50 mM ABC buffer (fresh 50 mM NH_4_HCO_3_ in water) by addition of 50 mM ABC buffer and centrifugation at 14,000*g* for 10 min four times. The filter unit is transferred to a new collection tube and 100 μl trypsin (0.1 μg μl^−1^ in 50 mM ABC buffer) is added to the sample, followed by incubation overnight at 37 °C. Tryptic peptides are collected from the filter unit by centrifugation at 14,000*g* for 10 min and addition of 100 mM NH_4_HCO_3_ buffer three times. Phenylmethylsulfonyl fluoride (1 mM final concentration in ethanol) is then added to the tryptic peptide solution to inactivate residual trypsin (4 °C for >2 h).

### N-glyco-FASP

The protocol for N-glyco-FASP was adapted from the protocol described by Zielinska *et al*.^24^. In an Ultracel YM-30 filter unit (Millipore, Billerica, MA), 100 μl of 2 μg μl^−1^ SNAP-Fbs1 GYR protein (in 20 mM Tris.HCl pH 7.5, 450 mM NaCl, 1 mM EDTA) or 100 μl of lectin mixture (90 μg ConA, 90 μg WGA and 71.5 μg RCA_120_ in 2.5 mM CaCl_2_, 2.5 mM MnCl_2_, 1.25 M NaCl, 50 mM Tris.HCl, pH 7.5) plus 50 μl of 2 μg μl^−1^ tryptic peptides from human serum (in 100 mM NH_4_HCO_3_) and 100 μl 100 mM NH_4_HCO_3_ were mixed and incubated without shaking at 4 °C for 1 h. After incubation, the filter unit was centrifuged at 14,000*g* for 10 min (or longer time until most of the liquid in the filter unit flowed through) at 4 °C to remove unbound peptides. The captured peptides in the filter unit were washed four times with 300 μl 100 mM NH_4_HCO_3_ and then eluted using 3 × 200 μl 50% formic acid. The eluted peptides were lyophilized to remove formic aid and NH_4_HCO_3_ and then used for downstream analyses including N-glycosite identification, N-glycan profiling and intact N-glycopeptide identification.

### LC-MS analysis

The LC-MS analysis were performed on a Thermo Dionex UltiMate HPLC system with a fluorescent detector, equipped with a Tosoh Amide 80 column (2.1 micron, 15 cm × 2.0 mm). The chromatographic conditions were as follows: solvent A, 100% acetonitrile; solvent B, ammonium formate, 50 mM, pH 4.4 in 100% water; flow rate, 350 μl min^−1^ throughout the run; gradient, started at 80% A, decreased to 40% from 2 to 34 min, before decreased further to 10% than back to 80% for reconditioning. The fluorescence detector was set at 350 nm for excitation and 420 nm for emission. The HPLC was coupled with a Thermo LTQ Orbitrap Velos mass spectrometer, equipped with a heated electrospray ionization interface. The major parameters were as follows: ESI spray voltage, 3.5 kV; capillary temperature, 250 °C; sheath gas, 11 psi; Aux gas and sweep gas flow rates, 0; S-lens RF level, 66%; Multiple 00 offset, 2.5 V; Lens 0 voltage, 6.5 V; Multiple 0 offset, 7.0 V; Lens 1 voltage, 16 V; Multiple 1 offset, 6.5 V; Multiple RF Amplitude, 600; Front lens, 7.75 V. The fluorescence traces of glycans released were superimposed for display. The identity of the major fluorescent peaks was assigned by corresponding mass value at the specific retention time, according to the glycan compositions of common mammalian N-glycan structures.

### N-glycan profiling from tryptic peptides of human

N-glycan release, hydrazide-mediated glycan clean-up, glycan 2-AB labelling and glycan solid phase extraction from 160 μg tryptic peptides with or without Fbs1 enrichment were performed according to the protocol described^55^. In brief, N-glycans released from N-glycopeptides using PNGase F were purified using Ultralink hydrazide resin (Thermo Fisher Scientific). The N-glycans were then labelled with 2-AB (Sigma) and extracted using HyperSep Diol SPE cartridges (Thermo Fisher Scientific). Each step was performed according to the manufacturer's protocol. 2-AB derivatized N-glycans were then separated by ultra-performance liquid chromatography (UPLC) with fluorescence detection on a Waters ACQUITY UPLC H-Class instrument consisting of a binary solvent manager, sample manager and fluorescence detector under the control of Empower 3 chromatography workstation software (Waters, Milford, MA). Glycans were separated using a Waters Ethylene Bridged Hybrid (BEH) Glycan column, (130A, 1.7 μm, 2.1 mm × 150 mm, Part no. 186004742) with 50 mM ammonium formate pH 4.4, as solvent A and acetonitrile as solvent B. The separation was performed using a linear gradient of 70–53% acetonitrile at 0.56 ml min^−1^ in 30 min. The 2-AB-labelled glycans were re-suspended in 20 μl of H_2_O and 5 μl was analysed by UPLC. An injection volume of 10 μl per sample from 16.7 μl 7:3 acetonitrile:H_2_O. Samples were maintained at 4 °C prior to injection and the separation temperature was 40 °C. The fluorescence detection excitation/emission wavelengths were λex=330 nm and λem=420 nm, respectively. The system was calibrated using an external standard of hydrolysed 2-AB-labelled glucose oligomers. Glucose units (GU) were assigned using a fifth-order polynomial distribution curve. Glycan structures were assigned to major peaks according to the glucose unit of each peak and by comparison to the published glycan structures determined using identical chromatographic conditions^56^.

### PNGase F deglycosylation in ^18^O water

Lyophilized peptides (100 μg tryptic peptide without Fbs1 enrichment or N-glycopeptides enriched by Fbs1 GYR from 100 μg tryptic peptide) were dissolved in 50 μl of 50 mM NH_4_HCO_3_ in ^18^O water and then incubated with 800 Unit PNGase F at 37 °C for 1 h. After deglycosylation, the samples were subjected to MS analysis immediately to reduce the chemical deamidation in ^18^O water.

### N-glycosite or intact N-glycopeptide identification

The peptides deglycosylated by PNGase F in ^18^O water or intact N-glycopeptides were subjected to LC-MS/MS. Easy nano-flow HPLC pump (Thermo Fisher Scientific) was used with the following buffer solutions: 0.1% formic acid in water (buffer A) and 0.1% formic acid in acetonitrile (buffer B). Samples were autosampler loaded directly onto an analytical column (25 cm Phenomenex Aqua 3 μm). Peptides were eluted using the following gradient: 2 min at 5%B, 120 min to 40%B, 5 min to 70%B, 5 min at 70%B, 2 min to 5%B, 6 min at 5%B. The flow rate was at 300 nl min^−1^ (splitless).

Peptides eluted from the microcapillary fritless column were directly electrosprayed into a quadrupole-orbitrap mass spectrometer (QExactive, Thermo Fisher Scientific). For ionization, 2500 V spray voltage, 250C capillary temperature were used with S-lens RF 60%. The quadrupole-orbitrap instrument was operated in the data-dependent mode and switched between full-scan MS and MS/MS acquisition. Full-scan MS (300–1,750 *m*/*z*) were acquired at resolution 140,000 (*m*/*z* 200) with AGC 1 × 10^6^ target value. HCD spectra were acquired for the 12 most intense ions (*z*≥2) at resolution 17,500 (*m*/*z* 200), 60 ms injection time and AGC 5 × 10^5^ target value. HCD peptide fragments were acquired at 27% normalized collision energy. Dynamic exclusion was set to 30 s.

### LC-MS/MS data analysis

Data analysis was performed using Byonic (Protein Metrics). For all data analysis workflows, protein false-discovery rate was set to 1%. Acquired spectra were searched against uniprot human proteome (Proteome ID: UP000005640) complemented with common contaminants (Fbs1 was also set as a contaminant). Trypsin specificity was allowed with up to two missed cleavages, carbamidomethyl (C) fixed and oxidation (M) variable. For N-glycosite analysis, incorporation of ^18^O during deamidation of asparagine to aspartic acid was considered as variable modification with mass shift of 2.988 Da. For the intact glycopeptide search, 57 human plasma N-glycan structures (from Byonic database) were considered as variable modification. Six naturally truncated or paucimannose N-glycan structures^57^ ((HexNAc(1), HexNAc(2), HexNAc(1)Fuc(1), HexNAc(2)Fuc(1), HexNAc(2)Hex(1) and HexNAc(2)Hex(2)) are included in this 57 plasma N-glycan structures.

### Data availability

The data supporting the findings of this study are available within the paper and its Supplementary Information files and available from the corresponding author upon request.

## Additional information

**How to cite this article:** Chen, M. *et al*. An engineered high-affinity Fbs1 carbohydrate binding protein for selective capture of N-glycans and N-glycopeptides. *Nat. Commun.* **8,** 15487 doi: 10.1038/ncomms15487 (2017).

**Publisher's note:** Springer Nature remains neutral with regard to jurisdictional claims in published maps and institutional affiliations.

## Supplementary Material

Supplementary Information Supplementary Figures

Supplementary Data 1: N-glycosite identification by PNGase F/18O water method in the pre-enrichment samples. Tab1: All MS spectra. The spectra with N[+2.988] are highlighted in yellow. Tab2: MS spectra with N[+2.988] in NXT/S motif. Tab3: MS spectra with N[+2.988] in non-NXT/S motif, Tab4: Identified unique N-glycosites. "-N-" represents N-glycosylation site. Tab5: Identified N-glycoproteins.

Supplementary Data 2: N-glycosite identification by PNGase F/18O water method in the Fbs1 GYR enrichment samples. Tab1: All MS spectra. The spectra with N[+2.988] are highlighted in yellow. Tab2: MS spectra with N[+2.988] in NXT/S motif. Tab3: MS spectra with N[+2.988] in non-NXT/S motif, Tab4: Identified unique N-glycosites. "-N-" represents N-glycosylation site. Tab5: Identified N-glycoproteins.

Supplementary Data 3: Intact N-glycopeptide identification in the pre-enrichment, and samples. Tab1: All MS spectra from three LC-MS/MS runs (highlighted by different colors for each run). Tab2: MS spectra of intact N-glycopeptide from three LC-MS/MS runs (highlighted by different colors for each run). Tab3: Organized intact N-glycopeptides. In Tab3, N-glycosite is indicated by N@; peptide mis-cleavage was manually removed; and Sodium in the N-glycan composition was removed. Data in Tab3 is the original source of Supplementary Data 6.

Supplementary Data 4: Intact N-glycopeptide identification in the lectin enrichment samples. Tab1: All MS spectra from three LC-MS/MS runs (highlighted by different colors for each run). Tab2: MS spectra of intact N-glycopeptide from three LC-MS/MS runs (highlighted by different colors for each run). Tab3: Organized intact N-glycopeptides. In Tab3, N-glycosite is indicated by N@; peptide mis-cleavage was manually removed; and Sodium in the N-glycan composition was removed. Data in Tab3 is the original source of Supplementary Data 6.

Supplementary Data 5: Intact N-glycopeptide identification in the Fbs1 GYR enrichment samples. Tab1: All MS spectra from three LC-MS/MS runs (highlighted by different colors for each run). Tab2: MS spectra of intact N-glycopeptide from three LC-MS/MS runs (highlighted by different colors for each run). Tab3: Organized intact N-glycopeptides. In Tab3, N-glycosite is indicated by N@; peptide mis-cleavage was manually removed; and Sodium in the N-glycan composition was removed. Data in Tab3 is the original source of Supplementary Data 6.

Supplementary Data 6, N-glycosylation micro-heterogeneity detected by intact N-glycopeptide identification using pre-enrichment, lectin enrichment, and Fbs1 GYR enrichment. This summary data was generated using Excel PivotTables from the data in Tab3 of Supplementary Data 3, 4, and 5. The orange row indicates N-glycoprotein identification. Underneath protein identification, N-glycosites (in green rows) are listed. Underneath each N-glycosite, N-glycan compositions (in red) attaching to this N-glycosite are listed. N@ indicates the asparagine with N-glycan attached. The light blue columns indicate the spectral counts of each N-glycoform.

Peer Review File
