# Supplementary material for: An engineered high affinity Fbs1 carbohydrate binding protein for selective capture of N-glycans and N-glycopeptides
Source: Nat Commun. 2017 May 23;8:15487. doi: 10.1038/ncomms15487 (PMC5457524; doi:10.1038/ncomms15487)
Supplement: Supplementary Information — Supplementary Figures [file ncomms15487-s1.pdf]

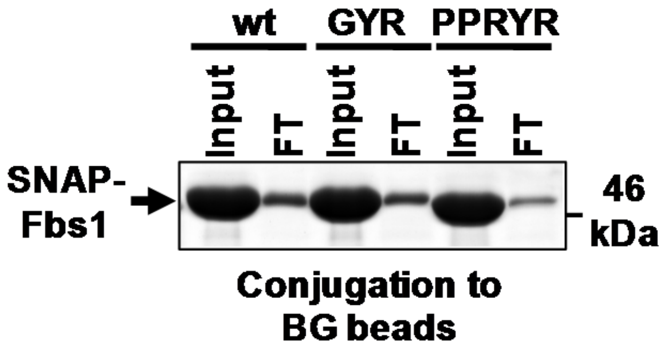

Supplementary Figure 1. Conjugation of wt, GYR or PPRYR Fbs1 protein to BG beads. FT (flow-through) denotes protein that was not conjugated to the BG beads. The conjugation efficiency can be estimated by the protein amount in the input lane minus the FT.

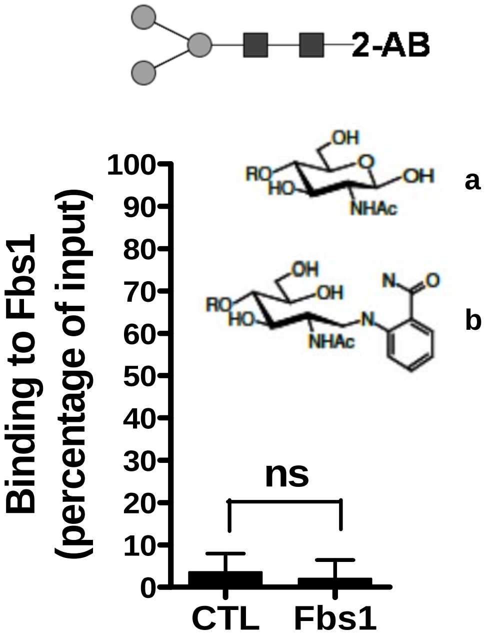

Supplementary Figure 2. 2-aminobenzamide (2-AB) fluorophore labeling at the reducing end of M3N2 abolishes binding by Fbs1 beads. M3N2-2-AB (structure shown at top) was incubated with Fbs1 beads. BG beads conjugated with β-lactamase were used as control beads (CTL). The binding affinity was measured as fluorescence retained on the beads: percentage of input equals fluorescence on the beads/input fluorescence. Chemical structures of the GlcNAc at the reducing end with or without 2-AB labeling are shown as “a” and “b”. ns denotes no statistical significant difference (p value 0.989, >0.05, t-test, two-tailed). Results represent the mean ± s.e.m. of four replicates.

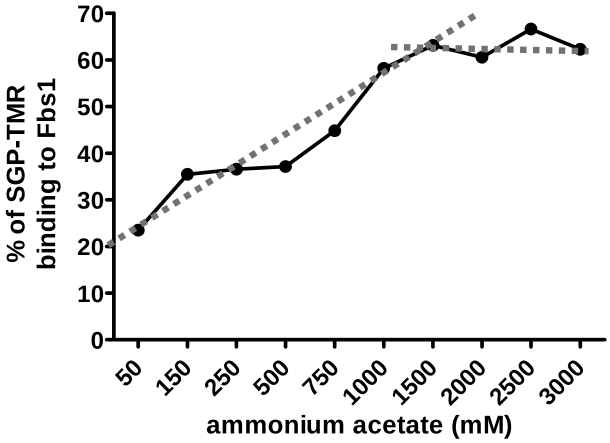

Supplementary Figure 3. Fbs1 binding to N-glycopeptides in high salt. The effect of increasing amount of salt (50-3000 mM ammonium acetate pH7.5) on binding of Fbs1 to SGP-TMR. Sloped dashed line indicates that SGP-TMR binding increases with increasing salt concentration (50 to 1500 mM), while the level dashed line indicates SGP-TMR binding reaches the maximum at 1500 to 3000 mM.

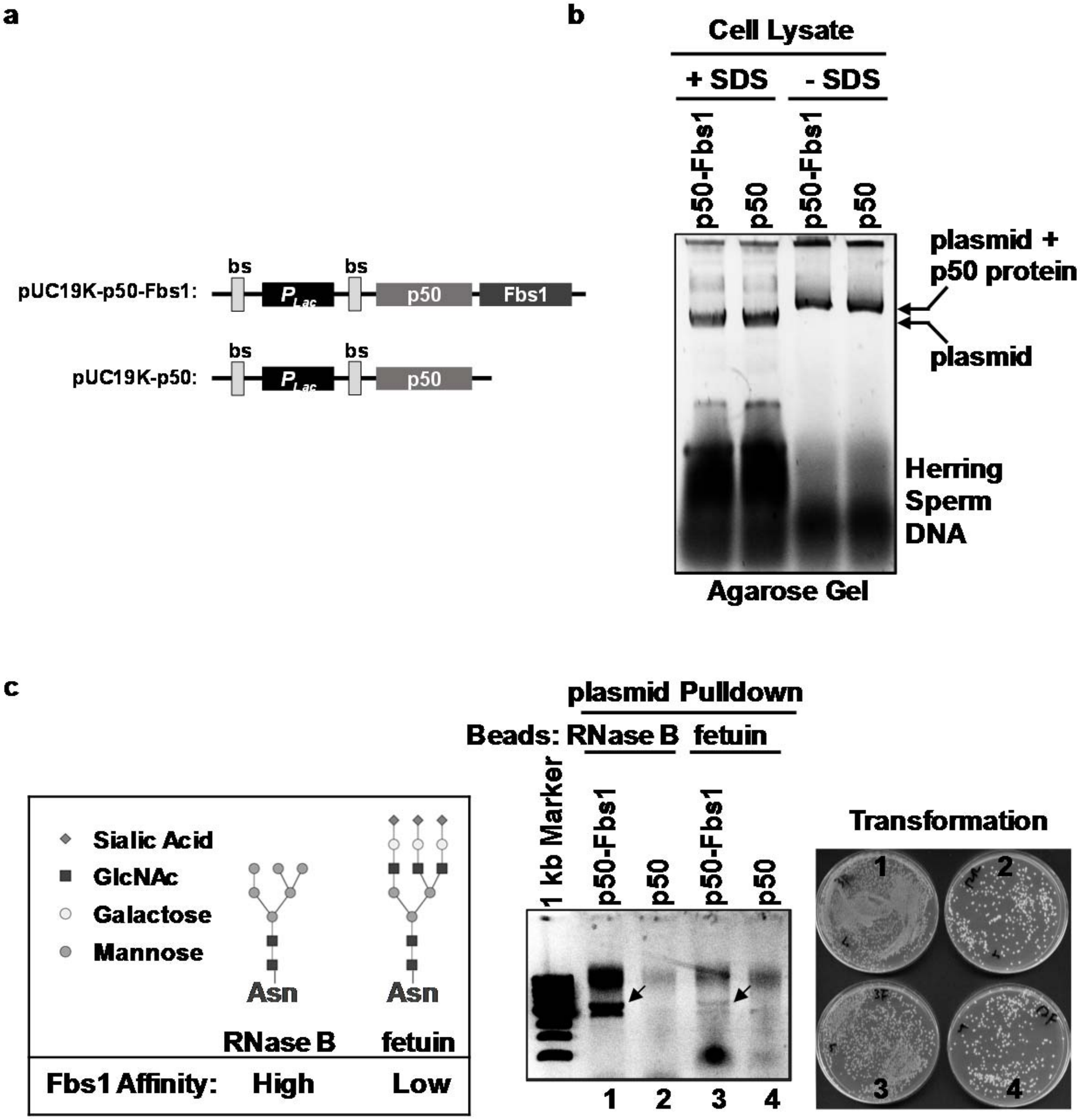

Supplementary Figure 4. The Fbs1 plasmid display system.

a. Illustration of the Fbs1 plasmid display constructs. pUC19K-p50-Fbs1 encoding the p50-Fbs1 fusion protein was used for Fbs1 display. pUC19K-p50 encoding p50 only was used as a control plasmid. Both plasmids contain p50 DNA binding sequences. P_{Lac} denotes the lactose-inducible promoter. bs denotes the p50 DNA binding site (5'-GGGAATTCCC-3').

b. p50-Fbs1 fusion protein binds to the encoding plasmid to link genotype and phenotype. *E. coli* cells harboring pUC19K-p50-Fbs1 or pUC19K-p50 were lysed using the spheroplast method described in Materials and Methods section. The cell lysates mixed with DNA loading buffer with or without SDS were analyzed by agarose gel electrophoresis. SDS was used to disrupt the interaction between the encoding plasmids and p50 or p50-Fbs1 proteins. The mobility shift (compare +/- SDS) indicates p50 protein or p50-Fbs1 fusion protein stably binds to their encoding plasmids. Herring sperm DNA is present due to carryover from the lysis buffer.

c. Left panel shows the glycan structures of RNase B and fetuin, and relative affinity to Fbs1. p50-Fbs1 protein and encoding plasmid DNA complexes were pulled down by either RNase B or fetuin beads. *E. coli* harboring pUC19K-p50-Fbs1 or pUC19K-p50 (as a control) were lysed using the spheroplast method described in the Materials and Methods section. The cell lysates were then subjected to RNase B and fetuin bead pulldown. The bound and eluted plasmid DNA was analyzed by agarose gel electrophoresis (middle panel). The bound and eluted pUC19K-p50-Fbs1 plasmid is indicated by arrows. The amount of isolated plasmid roughly correlates to the affinity of wt Fbs1 for the respective target proteins. The amount of isolated plasmid DNA was also confirmed by transformation into *E. coli* competent cells (right panel). The relative number of transformants (right panel) indicates that each in vitro selection/ transformation cycle may function to enrich for high affinity Fbs1 variants.

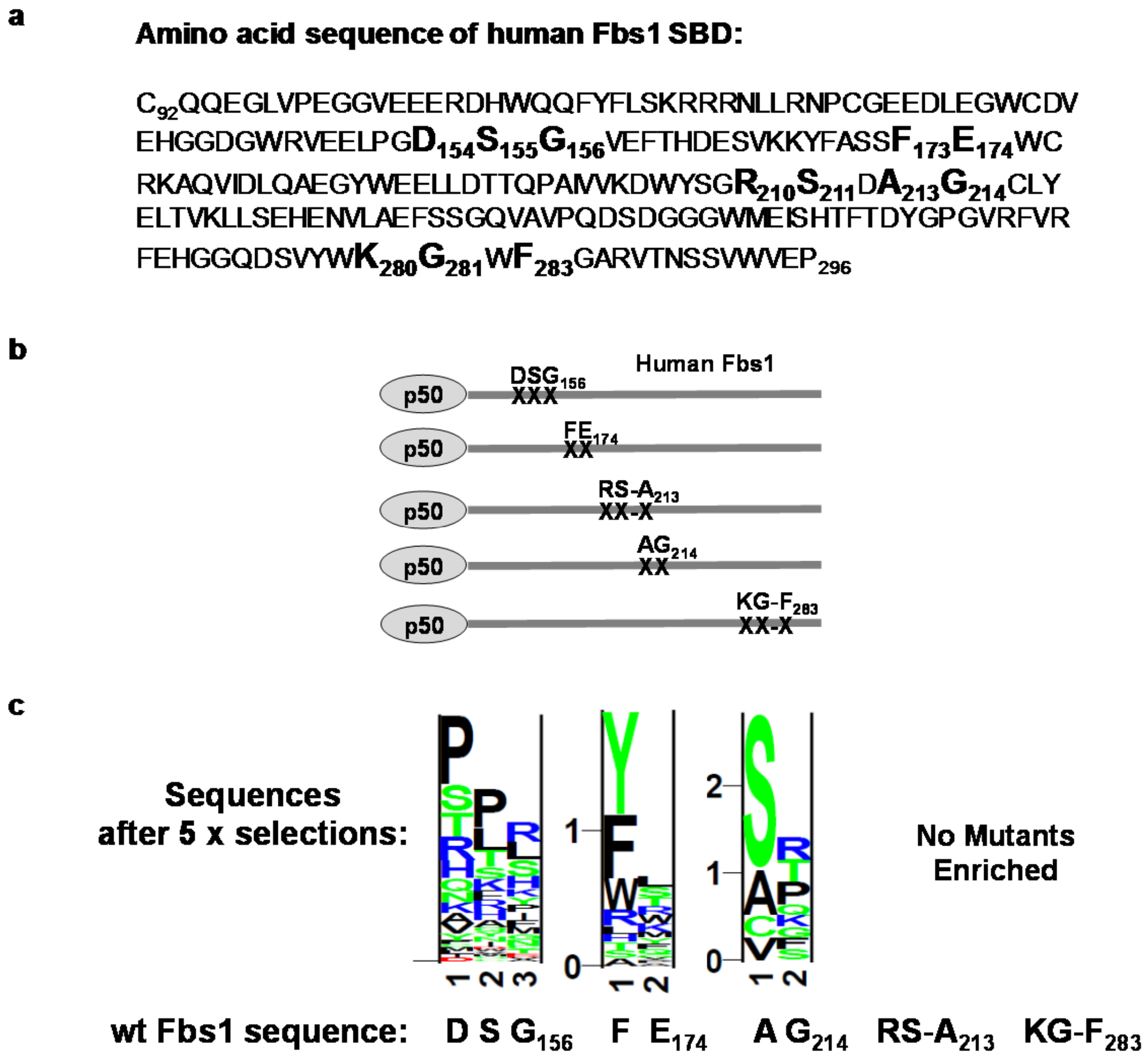

Supplementary Figure 5. Primary selection of Fbs1 mutants with higher affinity to fetuin by plasmid display.

a. Amino acid sequence of human Fbs1 sugar binding domain (SBD). The amino acid residues that were subjected to saturation mutagenesis are in bold. The numbering of amino acid residues is according to full-length human Fbs1.

b. Illustration of each saturation mutagenesis sub-library. The amino acid sequences of wt human Fbs1 are shown above the p50-Fbs1 fusion proteins, and “x” indicates codon positions subjected to mutagenesis. The last amino acid in each position is numbered according to the full-length wt human Fbs1.

c. Amino acid sequence logo indicating potential Fbs1 mutants with higher affinity to complex N-glycans. After 5 cycles of p50-Fbs1 plasmid display selection against fetuin beads, 121 clones were sequenced, and the amino acids in each mutagenesis position were plotted as a sequence logo to indicate the amino acid distribution frequency. The amino acid sequences in wt Fbs1 are shown under the corresponding logos.

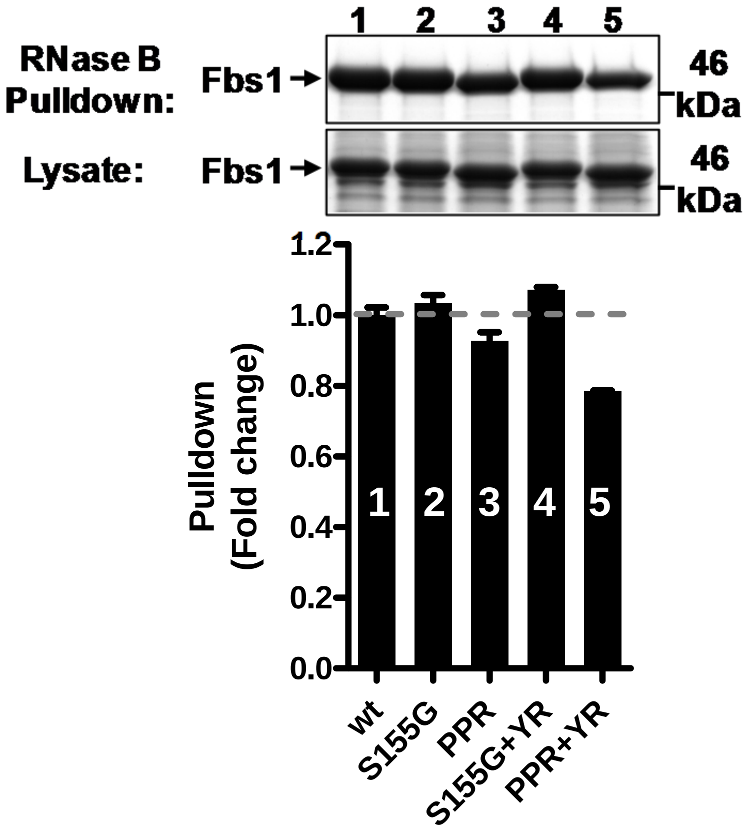

Supplementary Figure 6. Evaluation of Fbs1 variant binding affinity to RNase B. *E. coli* cell lysates containing the same amount of wt Fbs1 or Fbs1 variant proteins were subjected to an RNase B bead pulldown assay. The bound Fbs1 was analyzed by SDS-PAGE (upper panels) and quantified by ImageJ. The amount of bound Fbs1 variant protein was standardized to that of bound wt Fbs1 and the fold change was calculated (bar graph). The relative affinity to RNase B of each Fbs1 variant is indicated by the fold change. A representative SDS-PAGE gel is shown from two experiments.

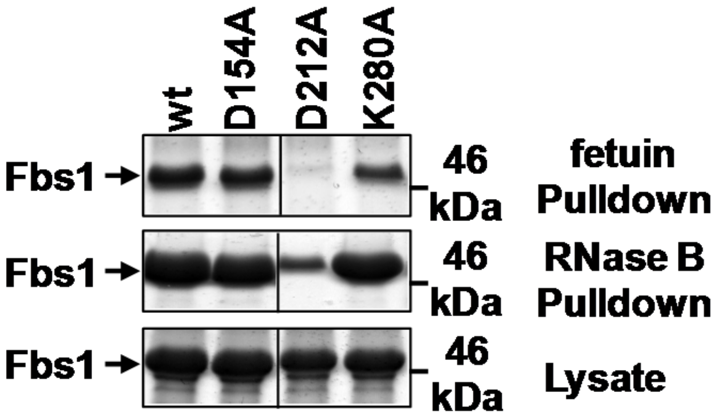

Supplementary Figure 7. Representative data: alanine scanning of Fbs1 glycan binding site. *E. coli* cell lysates containing wt, D154A, D212A or K280A Fbs1 protein were subjected to a fetuin or RNase B pulldown assay. The bound Fbs1 proteins were analyzed by SDS-PAGE. The amino acid numbering is according to full-length human Fbs1. A representative SDS-PAGE gel is shown from two experiments.

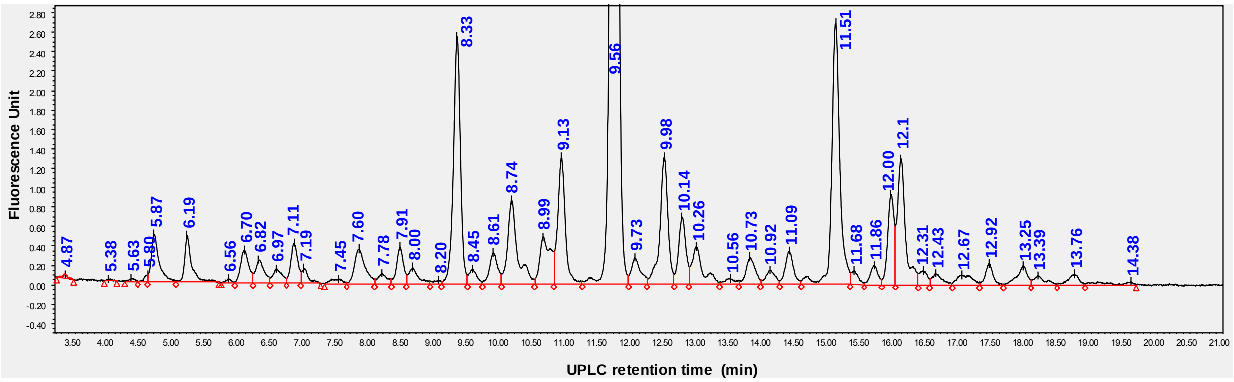

Supplementary Figure 8. Glucose unit (GU) assignment. GU values are assigned to each peak in the 2-AB-labeled N-glycan profile from the pre-enrichment sample of HSA-depleted human serum using a 2-AB-labled glucose homopolymer standard.

atgggcagcagccaccaccatcatcaccatggcgacaaagattgcgaaatgaaacgtaccaccctggatagcccgctgggcaaa
ctggaactgagcggctgcgaacagggcctgcatgaaattaaactgctgggtaaaggcaccagcgcggccgatgcggttgaagttcc
ggccccggccgccgtgctgggtggtccggaaccgctgatgcaggcgaccgcgtggctgaacgcgtattttcatcagccggaagcga
ttgaagaatttccggttccggcgctgcatcatccggtgtttcagcaggagagctttacccgtcaggtgctgtggaaactgctgaaagtggt
taaatttggcgaagtgattagctatcagcagctggcggccctggcgggtaatccggcggccaccgccgccgttaaaaccgcgctgag
cggtaacccggtgccgattctgattccgtgccatcgtgtggttagctctagcggtgcggttggcggttatgaaggtggtctggcggtgaa
agagtggctgctggcccatgaaggtcatcgtctgggtaaaccgggtctgggacctgcagctataggcgcgccaggatccggttctgg
ctctcctgcaggttgtcaacaagaaggtctggtcccggaaggtggtgtggaagaagaacgcgatcattggcaacagttttactttctga
gcaaacgccgtcgtaacctgctgcgcaatccgtgcggtgaagaagatctggaaggctggtgtgacgtcgaacatggcggtgatggtt
ggcgtgtggaagaactgccgggtgac**GGT**ggcgtggaatttacccacgatgaaagcgtgaaaaaatattttgcgagctct**TATC**
GTtggtgccgcaaagcgcaagtgattgacctgcaagccgaaggctactgggaagaactgctggataccacgcagccggcgatcg
tggttaaagactggtattcaggtcgttcggatgccggctgtctgtacgaactgacggttaaactgctgagcgaacatgaaaacgtcctg
gcagaatttagttccggtcaggtcgctgtgccgcaagatagtgacggcggtggctggatggaaatttcacataccttcacggattatggt
ccgggcgttcgttttgtccgcttcgaacacggtggccaagattccgtttactggaaaggctggtttggtgcccgtgtgacgaactcaagc
gtgtgggtggaaccgtaa

Supplementary Figure 9. DNA sequence of SNAP-Fbs1 GYR open reading frame. DNA sequences encoding 6his tag, SNAP tag, and Fbs1 are highlighted in yellow, gray, and blue, respectively. The GYR mutations are in bold/uppercase.
