# Supplementary material for: An engineered high affinity Fbs1 carbohydrate binding protein for selective capture of N-glycans and N-glycopeptides
Source: Nat Commun. 2017 May 23;8:15487. doi: 10.1038/ncomms15487 (PMC5457524; doi:10.1038/ncomms15487)
Supplement: Peer Review File [file ncomms15487-s8.pdf]

Editorial Note: This manuscript has been previously reviewed at another journal that is not operating a transparent peer review scheme. This document only contains reviewer comments and rebuttal letters for versions considered at Nature Communications.

PEER REVIEW FILE

Reviewers' Comments:

Reviewer #1 (Remarks to the Author):

The authors have adequately addressed all of the original concerns. However, as a necessary part of the transparency process, the authors should add the complete DNA sequence for Fbs1 GYR variant to the supplement so that their work can be reproduced by other labs.

Reviewer #2 (Remarks to the Author):

The authors have provided a detailed comparison of the performance of engineered Fbs1 verses conventional lectins for enrichment of glycopeptides from serum. They show convincing results that the engineered Fbs1 produces superior results in terms of quantity of N-glycopeptide enriched and specificity for N-glycans.
